# Physiological and transcriptomic analyses revealed gene networks involved in heightened resistance against tomato yellow leaf curl virus infection in salicylic acid and jasmonic acid treated tomato plants

**DOI:** 10.3389/fmicb.2022.970139

**Published:** 2022-09-14

**Authors:** Peng Wang, Sheng Sun, Kerang Liu, Rong Peng, Na Li, Bo Hu, Lumei Wang, Hehe Wang, Ahmed Jawaad Afzal, Xueqing Geng

**Affiliations:** ^1^College of Horticulture, Shanxi Agricultural University, Jinzhong, Shanxi, China; ^2^School of Agriculture and Biology, Shanghai Jiao Tong University, Shanghai, China; ^3^Institute of Quality and Safety Testing Center for Agro-Products, Xining, Qinghai, China; ^4^Edisto Research and Education Center, Clemson University, Blackville, SC, United States; ^5^Division of Science, New York University, Saadiyat Island Campus, Abu Dhabi, United Arab Emirates

**Keywords:** TYLCV, SA, JA, coronatine, tomato, RNA-seq

## Abstract

Tomato yellow leaf curl virus (TYLCV), a member of the genus *Begomovirus* of the *Geminiviridae* family, causes leaf curl disease of tomato that significantly affects tomato production worldwide. SA (salicylic acid), JA (jasmonic acid) or the JA mimetic, COR (coronatine) applied exogenously resulted in improved tomato resistance against TYLCV infection. When compared to mock treated tomato leaves, pretreatment with the three compounds followed by TYCLV stem infiltration also caused a greater accumulation of H_2_O_2_. We employed RNA-Seq (RNA sequencing) to identify DEGs (differentially expressed genes) induced by SA, JA, COR pre-treatments after Agro-inoculation of TYLCV in tomato. To obtain functional information on these DEGs, we annotated genes using gene ontology (GO) and Kyoto encyclopedia of genes and genomes (KEGG) databases. Based on our comparative analysis, differentially expressed genes related to cell wall metabolism, hormone signaling and secondary metabolism pathways were analyzed in compound treated samples. We also found that TYLCV levels were affected in *SlNPR1* and *SlCOI1* silenced plants. Interestingly, compared to the mock treated samples, SA signaling was hyper-activated in *SlCOI1* silenced plants which resulted in a significant reduction in viral titer, whereas in *SINPR1* silencing tomato plants, there was a 19-fold increase in viral load. Our results indicated that SA, JA, and COR had multiple impacts on defense modulation at the early stage of TYLCV infection. These results will help us better understand SA and JA induced defenses against viral invasion and provide a theoretical basis for breeding viral resistance into commercial tomato accessions.

## Introduction

Tomato yellow leaf curl virus (TYLCV), belonging to the genus *Begomovirus* of the *Geminiviridae* family, contains circular single-stranded DNA (ssDNA) which is 2.7–2.8 kb in length. As a destructive viral pathogen, TYLCV causes a myriad of host disease symptoms ranging from upward curling of leaves and chlorosis to a reduction in leaf size, resulting in significant yield losses (Prasad et al., 2020). In nature, the virus is spread by whiteflies (Huang et al., 2017), and the infection in tomato, as well as cucumber and pepper has been associated with the global spread of the pathogen (Li T. et al., 2019). The spread is further exacerbated due to the high migration rate and reproductive capacity of whiteflies.

Plant hormones play key regulatory roles in defense gene regulation (Du et al., 2017). JA (jasmonic acid) and SA (salicylic acid) are two important defense hormones implicated in the elicitation of plant defenses (Yu et al., 2015). SA plays a key role in resistance against biotrophic pathogens, and induces systemic acquired resistance (SAR) (Glazebrook, 2005; Pieterse et al., 2012, 2014). NPR1 (non-expressor of pathogenesis related genes 1) is one SA receptor that makes essential contributions to SA signaling in *Arabidopsis* (Wu et al., 2012; Ding et al., 2018). In A*rabidopsis*, three proteins (NPR1, NPR3, and NPR4) were proposed to function as SA receptors, as they were shown to bind to SA. NPR1 was proposed to function as a transcriptional co-activator, whereas NPR3/NPR4 were suggested to function as transcriptional co-repressors that induce NPR1 degradation. It was further shown that SA inhibits NPR3/NPR4 function to promote the expression of downstream immune regulators (Ding et al., 2018). On the other hand, JA signaling is activated when plants are attacked by necrotrophic pathogens or herbivores and leads to the activation of induced systemic resistance (ISR) (Pieterse et al., 2014). COI1 (CORONATINE INSENSITIVE1) is a key component of the JA signaling pathway. JA-Ile interacts with a complex of COI1 and jasmonate ZIM-domain transcriptional repressors protein to induce proteasome-mediated degradation of the JAZ protein by the SCF^*COI*1^ ubiquitin E3 ligase complex (Thines et al., 2007; Sheard et al., 2010). A previous study showed that the exogenous application of SA could inhibit viral accumulation and enhance the resistance response in susceptible tomato cultivars (Sade et al., 2014). The C4 protein from TYLCV can shift its localization from the plasma membrane to the chloroplast to inhibit SA dependent responses in tomato (Corrales-Gutierrez et al., 2020). Further, the ßC1 protein from the betasatellite of tomato yellow leaf curl China virus suppressed JA dependent plant terpene biosynthesis (Li et al., 2014) and the JA regulated biosynthesis of phytoalexin prevented the virus transmitting from the vector to the plant (Chen et al., 2019). These results show that both SA and JA signaling pathways are involved in plant defense regulation against TYLCV infection. However, the overall mechanism by which SA and JA signaling impact plant resistance to TYLCV are still elusive.

Coronatine (COR) produced by specific strains of *Pseudomonas syringae*, is a structural and functional mimic of the bioactive jasmonic acid conjugate JA-Ile and targets the JA-receptor COR-insensitive 1 (COI1) in *Arabidopsis* and tomato (Feys et al., 1994; Katsir et al., 2008; Geng et al., 2014). COR is more active than JA-Ile at stabilizing interactions between JAZ and COI1 protein in tomato (Thilmony et al., 2006; Katsir et al., 2008). COR also acts as a plant growth regulator to elicit the production of secondary metabolites including saponins and phytoalexins (Tamogami and Kodama, 2000; Lee et al., 2018). It has been proposed that the exogenous application COR may promote abiotic resistance in plants (Xie et al., 2015).

In the current study, we discovered that the pre-treatment of tomato plant with SA, JA, and COR followed by Agro-inoculation of TYLCV could decrease TYLCV accumulation at the early stage of infection. DAB staining showed that TYLCV invasion that followed the three treatments resulted in a higher level of H_2_O_2_ accumulation when compared to mock treatment. We investigated the transcriptome of tomato plants infected by TYLCV under these three treatments. Gene ontology (GO) terms and Kyoto Encyclopedia of Genes and Genomes (KEGG) databases are routinely used to annotate functions of differentially expressed genes (DEGs) in tomato plants. We focused on analysis of genes involved in cell wall biosynthesis as previous reports had shown that tomato leaves infected with TYLCV induced thickening of cell walls (Montasser et al., 2012). We also sought to identify differentially expressed genes in hormone signaling pathways and secondary metabolite regulation. In addition, we silenced *SlNPR1*, which is a central component in SA signal transduction, and *SlCOI1*, which functions as a JA-Ile receptor in order to further determine the roles of SA and JA signaling in TYLCV infection. Our results indicated that SA, JA and COR significantly altered defense signaling during the early stage of TYLCV infection and broaden our understanding of SA and JA signaling pathways involved in mediating resistance to TYLCV in tomato.

## Materials and methods

### Plant materials

The seeds of Tomato (*Solanum lycopersicum* var. Moneymaker) (susceptible to TYLCV infection) were germinated at 25°C, and then transferred to flats containing soil. Seedlings at the four-leaf stage were moved into plastic pots with soil and grown in a plant growth chamber under a 12 h light (320 μmol⋅m^–2^ s^–1^, 26°C)/12 h dark (18°C) cycle. The relative humidity was maintained between 60 and 70%.

### Treatments and tomato yellow leaf curl virus infection

Tomato plants containing five to six leaves after 3 weeks of growth were randomly divided into four groups. Solutions of JA (0.5 mM), SA (0.5 mM), COR (0.1 μM), and deionized water (mock) mixed with 0.04% silwet L-77 separately, were exogenously sprayed onto tomato leaves. The TYLCV infectious clone was provided by Professor Zhou Xueping of Zhejiang University (Zhang et al., 2009). After 24 h, all the seedlings were inoculated with *Agrobacterium* (OD_600_ = 1.0) carrying TYLCV (the clone was introduced into *Agrobacterium* GV3101) as described previously (Zhang et al., 2009). We injected 1 ml of solution into the plant stem, 5 cm above ground at three points by using a 1.0 ml syringe. Virus infection symptoms were determined visually by observing changes in leaf color and viral infection was confirmed through qPCR. Seven days post inoculation, samples from upper leaves were collected from the same nodes from both three compounds and mock treated plants. One hundred milligrams from each sample was taken for RNA-seq (RNA sequencing) and RT-qPCR verification. Each experiment included three biological replicates.

### Detection of tomato yellow leaf curl virus content in tomato plants

Quantitative PCR (qPCR) was used to detect relative TYLCV content from leaves of infected tomato plants (Sade et al., 2014, 2020; Li et al., 2017). All leaf samples from the infected plants were collected and subsequently frozen in liquid N_2_. Total DNA was extracted from the leaves using a DNA extraction kit (DNA secure Plant Kit, Shenggong, China). qPCR was carried out in the presence of SYBR Green (TaKaRa, Shanghai, China) by using a RealTime PCR system (Bio-Rad, CA, United States). The tomato *SlACTIN* gene (LOC101260631) (Zhang et al., 2021) was used as the normalizing control. The qPCR reaction was carried out as follows: 30 s at 94°C, followed by 40 cycles consisting of 10 s at 94°C, 30 s at 58°C, and 20 s at 72°C. The specificity of the amplified products was based on melting curves that were generated from 65 to 95°C with increments of 0.5°C/cycle. The melting curve data was analyzed by using the CFX Manager™ software (version 3.0, Bio-Rad, CA, United States). The primers used for detecting TYLCV were: F (5′-CACTCAATTCAGGCAGTAAATCC-3′), and R (5′-GACCCACTCTTCAAGTTCATCTG-3′). The primers used for *SlACTIN* are listed in Table 1. The TYLCV content in the mock treated leaves was set to 1. Shown are the mean and SEM from three independent biological replicates.

**TABLE 1:** Primers used in this manuscript.

Gene/Gene number	Forward	Reverse	Product length
*SlACTIN*	GATGGTGGGTATGGGTCAAA	AGGGGCTTCAGTTAGGAGGA	199
*SlNPR1*	GGTCAGTGTGCTCGCCTAT	TGAAAGGTAAAGGATGCGT	150
*SlCOI1*	AAGCACTTCATTTTCGTAGA	TCAGTAGAAAATCCAGAACAC	118
LOC101264227	GACATGTGGAAGGAAGCTTGG	ATCTCCAATTTCACTATCCAA	111
LOC101267111	AGCAGCTTCTTACATGTCAAC	TGGTTATGAGAACACGATCAA	116
LOC101256271	AAAACTCAACGATGGTGCAGC	GGCGTGTGGAAATTAGGCACT	94
LOC101243656	ACTTGGTGGTGAAACATTGAC	GCCCTAGCATCCTCACAAAGC	99
LOC101245298	GTCAAGCCTTTTGGGTTATCG	AGGATCCACTTCGTTCATCAT	134
LOC104648161	CAAATGCATGTCCCTTGTGTT	CAGATGAAAATCTACCTGACG	142
LOC101258353	GTTACTTTGTGCTTCAGCCAA	TCGGAGAGTGAGCTGGTGAGT	138
LOC101265701	AAACTCATTGAAAGACTTCTC	CCGGCATACTATAATCTGTAA	133
LOC101265854	GCTCGTGGTCAAGTCGGGGTT	GACCAGAATGAATCAAGTTGC	112
LOC109119038	GATGGCTTCACACTTCCCAAG	TACTCTCCATTTTGGTATCCC	141
LOC101256422	AGCCTTCCCATCTCATCATAC	TTGGGCCAATAGCTACACAAG	62
LOC109120532	ATGGACCCCGTAGAGCTAAAT	TGTGTATTCCCAAGCTTTGCA	109

### Detection of H_2_O_2_ accumulation in the leaves of tomatoes

24 hours post salicylic acid, jasmonate acid, and coronatine treatments 24, plants were inoculated with *Agrobacterium* (OD_600_ = 1.0) carrying TYLCV. After 7 days, leaves were collected to detect H_2_O_2_ accumulation by staining with 3,3-diaminobenzidine (DAB). In brief, leaves were immersed in 0.1% DAB solution and incubated at room temperature for 8 h. Leaf tissues were subsequently immersed in 95% ethanol and boiled for 15 min until the leaves were transparent. For every treatment, 3–4 leaves were collected. Each experiment included three biological replicates. A total of nine slides were made for each treatment and one slide was selected to represent the results.

### Transcriptome sequencing and library construction

Leaf samples from the four groups (mock treatment, JA treatment, SA treatment, and COR treatment) were collected at 7 days post TYLCV clone infection. The harvested samples were immediately frozen in liquid nitrogen and stored at −80°C. Total RNA was extracted with Trizol reagent (Invitrogen, Carlsbad, CA, United States). The concentration of RNA was determined using a NanoDrop spectrophotometer and the quality of RNA was assessed by resolving RNA on a 1.5% (w/v) agarose gel (Gao et al., 2015).

The mRNA was enriched from a pool of total RNA through Oligo-dT-enriched magnetic beads (Vazyme Biotech, Jiangsu, China). The enriched mRNA was sheared into short pieces by using an RNA fragmentation kit according to manufacturer’s instruction (Illumina, San Diego, CA, United States). The fragments were reverse-transcribed into single stranded cDNA with random N6 primers. The cDNA fragments were subjected to end repair and ligated with adapters, followed by PCR amplification. The amplified PCR products were used to generate the cDNA library. cDNA sequencing was carried out on the Huada Gene Technology BGISEQ-500 platform (Wuhan, Hubei, China).

### Read mapping, quality control, and functional annotation

We obtained raw data from the BGISEQ-500 platform. If more than half of the component bases in the reads had a quality score of <15, they were annotated as low quality reads and filtered by using Soapnuk (v1.5.2) software. Other low-quality regions such as adapter sequences and regions with an excessive number of unknown bases (annotated as “Ns”) were also filtered by Soap nuke (Li et al., 2008). The remaining clean reads were mapped onto the reference genome sequence (GCF_000188115.4_SL3.0)^1^ using the Hierarchical Indexing for Spliced Alignment of Transcripts HISAT2 (v2.0.4) system (Gao et al., 2014; Kim et al., 2015). Bowtie2 was used to align clean reads to the reference coding genomic sequence and the FPKM (fragments per kilobase of transcript per million mapped reads) was calculated by RSEM (v1.2.12) (Li and Dewey, 2011). Differentially expressed genes (DEGs) were identified by DEseq2 (v1.4.5) (Love et al., 2014), with the following set of conditions (| log_2_ Fold Change| ≥ 1, and a False discovery rate (FDR) <0.001. GO (gene ontology) annotation^2^ and KEGG (Kyoto Encyclopedia of Genes and Genomes) pathway enrichment^3^ of DEGs was used to gain insight into the functional classes and pathway information of the DEGs. The RNA-seq data for SA-treated sample (accession no: SRR19591763), JA-treated sample (accession no: SRR19591764), COR-treated sample (accession no: SRR19591765), and mock sample (accession no: SRR19591766) are available at the NCBI gene expression omnibus server.^4^

### Validation of candidate genes with virus induced gene silencing

VIGS (virus induced gene silencing) assays were performed as described (Yu et al., 2019). Briefly, the TRV-mediated VIGS system was used to silence two genes (*SlNPR1* and *SlCOI1*). Fragments of *SlNPR1* and *SlCOI1* amplified using specific primers were inserted into pTRV2 vectors. The constructs were introduced into *Agrobacterium* GV3101 by electroporation and injected into fully expanded leaves of 2-week-old tomato plants (Li et al., 2013). pTRV1 plus empty pTRV2 served as the negative control, whereas pTRV1 plus pTRV2-phytoene desaturase (*SlPDS*) served as the positive control. Independent cultures of *Agrobacterium* GV3101 carrying pTRV1, pTRV2, pTRV2-*SlNPR1*, pTRV2-*SlCOI1*, and pTRV2-*SlPDS* were liquid-cultured overnight in LB medium separately. Cultures were resuspended in 10 mM MgCl_2_ to a final OD_600_ = 1.0. Cultures were mixed with a 1:1 ratio. Approximately 1 ml of this suspension was used to inoculate the leaves of tomato plants. Silencing frequency (%) and silencing efficiency were calculated as described previously (Dinesh-Kumar et al., 2003). The primers used for amplification of *SlNPR1* and *SlCOI1* are listed in Table 1.

### Validation of differentially expressed genes with quantitative real-time RT-PCR

To access the reliability of our transcriptome data, we determined the expression profile of 12 randomly selected genes through quantitative real time PCR. RNA was extracted using the Trizol reagent (Invitrogen, United States) whereas first-stand cDNA synthesis was carried out using the PrimeScript™ RT and the gDNA Eraser (TaKaRa, Shanghai, China) kits. Quantitative real-time RT-PCR (qRT-PCR) was carried out on a RealTime PCR System (Bio-Rad, CA, United States) using the SYBR Premix Ex Taq II kit (Takara, China). The following reaction conditions were used: pre-denaturation at 95°C for 30 s, denaturation at 95°C for 5 s, extension at 60°C for 30 s for a total of 40 cycles. Relative gene expression was monitored in realtime and measured using the 2^–ΔΔCT^ method as previously described (Gao and Chen, 2018; Zhang et al., 2021). The gene LOC101260631, which encodes *SlACTIN* was used as the internal reference. qRT-PCR primer sequences for the 12 selected genes are shown in Table 1. Each treatment employed three independent biological replicates.

## Results

### The relative tomato yellow leaf curl virus content reduced in tomato plants under treatments of jasmonate acid, salicylic acid, and coronatine

**FIGURE 1:**
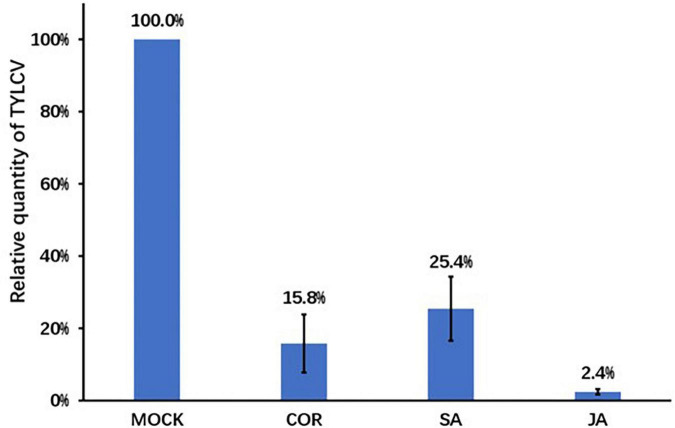
The relative tomato yellow leaf curl virus (TYLCV) contents in tomato leaves. Pre-treatment of coronatine (COR) (0.1 μM), salicylic acid (SA) (0.5 mM), jasmonate acid (JA) (0.5 mM), or mock was followed by the inoculation of the TYLCV infectious clones. Leaf samples were collected 7 days post infection. TYLCV levels were determined using qPCR and the TYLCV levels in the mock leaves were set to 1. Shown are the mean and SEM from three independent biological replicates.

We treated tomato plants with solutions of COR, SA, JA, or mock through foliar spray. Twenty-four hours post spray, we inoculated plants with the TYLCV infectious clone and detected TYLCV titer 7 days post infection. The TYLCV levels in compound treated leaves were significantly lower than those in mock treated leaves. Compared to the mock treatment, JA treatment caused a 50-fold reduction in TYLCV accumulation. COR treatment resulted in a 10-fold reduction, whereas SA treatment resulted in a 4-fold reduction in virus content (Figure 1). A typical symptom of TYLCV infection is leaf curling, which we observed 7 days post virus inoculation. The extent of curling of leaves for all three treatments was lower when compared with mock treated leaves (data not showed). These results indicated that COR, SA, and JA induced tomato defense responses to inhibit TYLCV accumulation at the early stage of infection.

### Reactive oxygen species production of tomato leaves under the exogenous application of jasmonate acid, salicylic acid, and coronatine

**FIGURE 2:**
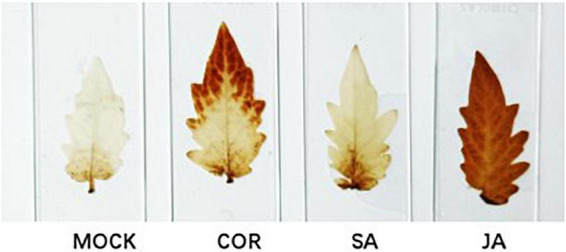
Detection of H_2_O_2_ by 3,3-diaminobenzidine (DAB) staining. Leaf samples were collected 7 days after inoculation with the tomato yellow leaf curl virus (TYLCV) infectious clone that were pre-treated with coronatine (COR), salicylic acid (SA), jasmonate acid (JA), or mock. Brown color in the leaves serves as a proxy for H_2_O_2_ production. Shown are representative pictures from three biological repeats.

ROS (reactive oxygen species) production is an early plant response to biotic and abiotic stress (O’Brien et al., 2012). ROS, especially H_2_O_2_, impacts plant tolerance through regulating signal recognition and transduction. In this study, we used 3,3-diaminobenzidine (DAB) staining to detect the accumulation of H_2_O_2_ 7 days after the Agro-inoculation of the TYLCV infectious clone. JA treatment resulted in robust accumulation of H_2_O_2_, whereas SA and COR treatments resulted in the accumulation of hydrogen peroxide, albeit to reduced levels (Figure 2). As a signaling molecule that activates defense responses against virus infection, H_2_O_2_ accumulation in JA treated samples showed the greatest reduction in TYLCV (Figure 1).

### Overview of the *Solanum lycopersicum* transcriptome

**FIGURE 3:**
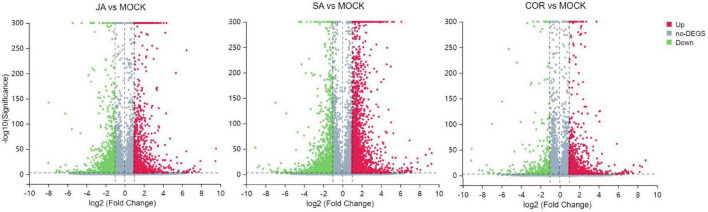
Volcano plots for differentially expressed genes (DEGs) including jasmonate acid (JA) treatment vs. mock treatment, salicylic acid (SA) treatment vs. mock treatment and coronatine (COR) treatment vs. mock treatment. The log_2_ fold change (*X*-axis) is plotted against the log_10_FDR (*Y*-axis). Red dots and green dots represent DEGs that were either up-regulated or down-regulated. Gray dots denote genes that were not significantly different between treatments and mock.

Next-Generation RNA sequencing (RNA-Seq) is a powerful tool used to study global transcriptional changes under biotic stress. In order to ascertain gene expression changes in tomato plants against TYLCV under the exogenous application of JA, SA, and COR, we performed transcriptome analysis on 12 samples. The transcriptome data was generated using DNBSEQ. After filtering out low-quality reads with SOAP nuke (v1.5.2) (Li et al., 2008), we obtained an average of 23.59 M high-quality reads per sample, accounting for >93% of the raw reads per sample (Table 2). The clean reads with a quality value of Q30 accounted for >94% of the total reads and a quality value of Q20 accounted for >98% of total clean reads (Table 2). The clean data were aligned to the genome sequence of S. *lycopersicum* (GCF_000188115.3_SL2.50)^5^ using the Hierarchical Indexing for Spliced Alignment of Transcripts (HISAT) system. More than 96% of reads were successfully mapped to the reference genome and the unique mapping rate for each sample was over 90%, indicating that the RNA-Seq data was of high quality. Based on transcriptome analysis, DEGs (differentially expressed genes) (| log_2_ Fold Change| ≥ 1, FDR < 0.001) from different comparative groups were identified. Briefly, a total number of 2,574 (958 up-regulated and 1,616 down-regulated), 3,864 (2,170 up-regulated and 1,694 down-regulated) and 1,406 DEGs (993 up-regulated and 413 down-regulated) genes were identified in JA vs. mock, SA vs. mock, and COR vs. mock comparisons. To make the visualization more intuitive, we have drawn a volcanic map to show the distribution of DEGs under three different treatments (Figure 3).

**TABLE 2:** Data mapped quality of the transcriptome.

Sample	Total raw reads (M)	Total clean reads (M)	Clean reads Q20 (%)	Clean reads Q30 (%)	Clean reads ratio (%)	Total mapping (%)
MOCK1	25.91	24.20	98.82	95.41	93.43	97.10
MOCK2	23.75	23.65	98.63	94.65	99.59	96.59
MOCK3	23.75	23.61	98.73	95.06	99.42	97.13
JA_T1	23.75	23.60	98.74	95.04	99.40	96.71
JA_T2	23.75	23.60	98.71	94.95	99.40	96.86
JA_T3	23.75	23.61	98.52	94.19	99.44	96.69
SA_T1	23.75	23.65	98.65	94.71	99.60	97.18
SA_T2	23.92	23.75	98.44	95.25	99.29	97.42
SA_T3	23.75	23.66	98.59	94.50	99.63	97.15
COR_T1	24.09	22.51	98.78	95.29	93.46	97.17
COR_T2	23.92	23.61	98.39	95.35	98.69	96.81
COR_T3	23.75	23.67	98.63	94.60	99.67	97.00

### Gene ontology analysis of differentially expressed genes

**FIGURE 4:**
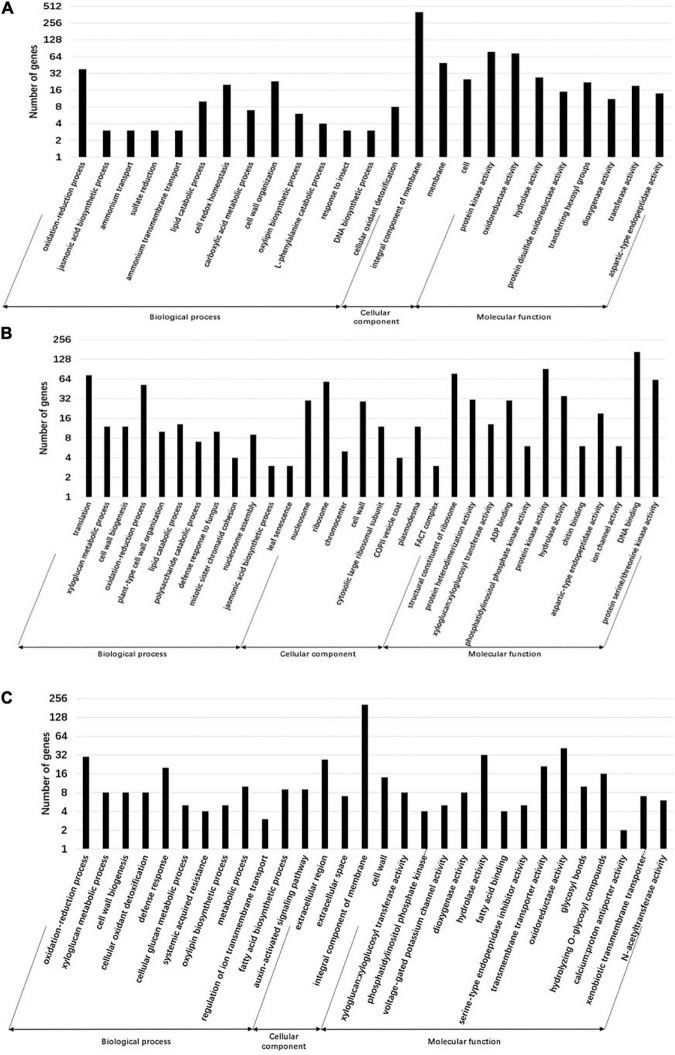
Gene ontology (GO) annotation of differentially expressed genes (DEGs) for different treatments. Genes are divided into the following three major functional categories: biological process, cellular component, and molecular function. The bars represent the enriched gene numbers in major sub-categories within each GO category. **(A)** GO classifications of differential genes in the jasmonate acid (JA) vs. mock group. **(B)** GO classifications of differential genes in the salicylic acid (SA) vs. mock group. **(C)** GO classifications of differential genes in the coronatine (COR) vs. mock group.

Gene ontology is a system of standardized scientific classification used to systematically annotate gene function. To determine the potential function of tomato DEGs involved in TYLCV infection under the exogenous application of JA, SA, and COR, we performed GO analysis. Our aim was to decipher potential biological processes and genes regulated under the three treatments. A total of 25, 32, and 30 GO terms were significantly enriched with DEGs regulated by JA, SA and COR respectively (*P* < 0.01) (Figure 4). Intriguingly, there were commonly enriched GO terms for both up-regulated and down-regulated DEGs among the three treatments. Up-regulated DEGs were commonly enriched in GO terms such as oxylipin biosynthetic process (GO:0031408), defense response (GO:0006952), and oxidation-reduction process (GO:0055114), while down-regulated DEGs were commonly enriched in terms such as auxin-activated signaling pathway (GO:0009734), plant-type cell wall organization (GO:0009664), and DNA replication initiation (GO:0006270). Moreover, DEGs from the three treatments also enriched for the “cell wall” category. This is not surprising as the first physical and defensive barrier against pathogens, the plant cell wall undergoes dynamic structural changes to prevent pathogen ingress (Nafisi et al., 2015).

### Important Kyoto encyclopedia of genes and genomes pathways influenced by the three treatments

**FIGURE 5:**
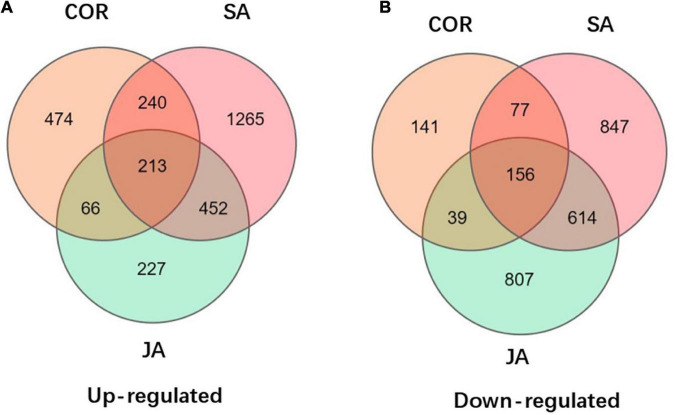
Venn diagrams displays the distribution of up-regulated and down-regulated differentially expressed genes (DEGs) upon coronatine (COR), salicylic acid (SA), and jasmonate acid (JA) treatments followed by tomato yellow leaf curl virus (TYLCV) invasion. **(A)** Up-regulated of DEGs by JA, SA, and COR treatments followed by TYLCV invasion. **(B)** Down-regulated of DEGs by JA, SA, and COR treatments followed by TYLCV invasion.

The KEGG database is a very useful resource for understanding high-level function of a biological system. To better understand the systemic roles of the three treatments, we also performed KEGG pathway enrichment analysis on the identified DEGs. There was a significant enrichment in 15, 11, and 19 KEGG pathways (*P* < 0.05) for DEGs induced by COR, SA and JA, respectively (Tables 3, 4). We identified 213 shared DEGs which were up-regulated in the three treatment groups (Figure 5A and Supplementary Table 1). Three pathways, including “Plant-pathogen interaction” (ko04626), “alpha-Linolenic acid metabolism” (ko00592), and “Phenylpropanoid biosynthesis” (ko00940) were the mostly enriched pathways on the basis of up-regulation. Meanwhile, there were 156 shared DEGs which were all down-regulated by the three treatments (Figure 5B and Supplementary Table 2) and significantly enriched for the “Plant hormone signal transduction” (ko04075) pathway. In particular, up-regulated DEGs induced by JA were enriched in many metabolic pathways such as “Tyrosine metabolism” (ko00350), and “Phenylalanine metabolism” (ko00360), which will be further characterized later. These results indicated that while the three treatments mostly induce different DEGs, they do share some common genes/pathways in response to TYLCV invasion.

**TABLE 3:** The Kyoto encyclopedia of genes and genomes (KEGG) pathways (top 10) enriched by up-regulated differentially expressed genes (DEGs) under the three treatments.

Treatment	Pathway ID	KEGG pathway	Gene number	*P*-value
JA	00940	Phenylpropanoid biosynthesis	28	4.52E-08
	04626	Plant-pathogen interaction	24	5.42E-06
	00945	Stilbenoid, diarylheptanoid, and gingerol biosynthesis	10	1.18E-05
	00941	Flavonoid biosynthesis	10	1.05E-04
	00592	alpha-Linolenic acid metabolism	9	1.76E-04
	00350	Tyrosine metabolism	7	0.003754
	00280	Valine, leucine, and isoleucine degradation	8	0.003862
	00591	Linoleic acid metabolism	4	0.005518
	00480	Glutathione metabolism	11	0.00604
	00071	Fatty acid degradation	7	0.008333
SA	04626	Plant-pathogen interaction	45	1.84E-08
	04016	MAPK signaling pathway-plant	35	4.98E-06
	00592	alpha-Linolenic acid metabolism	12	0.001178
	00562	Inositol phosphate metabolism	15	0.002055
	00565	Ether lipid metabolism	8	0.002609
	04075	Plant hormone signal transduction	39	0.003307
	04070	Phosphatidylinositol signaling system	14	0.005498
	00280	Valine, leucine, and isoleucine degradation	12	0.008001
	00564	Glycerophospholipid metabolism	16	0.013576
	00591	Linoleic acid metabolism	5	0.017725
COR	00500	Starch and sucrose metabolism	16	2.00E-04
	04016	MAPK signaling pathway-plant	17	0.001028
	04626	Plant-pathogen interaction	19	0.001129
	00908	Zeatin biosynthesis	8	0.005088
	00591	Linoleic acid metabolism	4	0.005518
	00950	Isoquinoline alkaloid biosynthesis	5	0.005555
	00053	Ascorbate and aldarate metabolism	7	0.006927
	00360	Phenylalanine metabolism	7	0.007606
	00562	Inositol phosphate metabolism	8	0.011395
	00350	Tyrosine metabolism	6	0.015016

**TABLE 4:** The Kyoto encyclopedia of genes and genomes (KEGG) pathways (top 10) enriched by down-regulated differentially expressed genes (DEGs) under the three treatments.

Treatment	Pathway ID	KEGG pathway	Gene number	*P*-value
JA	04075	Plant hormone signal transduction	36	3.18E-06
	00860	Porphyrin and chlorophyll metabolism	10	2.25E-04
	03030	DNA replication	10	3.19E-04
	00500	Starch and sucrose metabolism	19	3.65E-04
	04141	Protein processing in endoplasmic reticulum	24	0.001211
	00906	Carotenoid biosynthesis	6	0.015049
	00944	Flavone and flavonol biosynthesis	2	0.016211
	00591	Linoleic acid metabolism	4	0.016937
	00904	Diterpenoid biosynthesis	5	0.034046
	00920	Sulfur metabolism	5	0.042292
SA	03010	Ribosome	103	2.08E-40
	03030	DNA replication	12	3.67E-04
	00100	Steroid biosynthesis	10	4.34E-04
	00941	Flavonoid biosynthesis	11	0.006102
	00860	Porphyrin and chlorophyll metabolism	8	0.031731
	00944	Flavone and flavonol biosynthesis	2	0.031943
	00780	Biotin metabolism	4	0.06257
	00904	Diterpenoid biosynthesis	5	0.116215
	00920	Sulfur metabolism	5	0.139336
	00942	Anthocyanin biosynthesis	1	0.148024
COR	04075	Plant hormone signal transduction	23	8.96E-10
	00280	Valine, leucine and isoleucine degradation	6	9.85E-04
	00073	Cutin, suberine, and wax biosynthesis	4	0.002791
	00640	Propanoate metabolism	4	0.010436
	00904	Diterpenoid biosynthesis	3	0.021341
	04146	Peroxisome	5	0.024415
	00100	Steroid biosynthesis	3	0.028613
	00591	Linoleic acid metabolism	2	0.043216
	00900	Terpenoid backbone biosynthesis	3	0.081637
	00565	Ether lipid metabolism	2	0.09718

### Differentially expressed genes related to cell wall synthesis regulated by salicylic acid, jasmonate acid, and coronatine

Pathogen attack can result in plant cell wall fortification which prevents further pathogen ingress (O’Brien et al., 2012). Polysaccharides are the main component of plant cell walls. In our study, 25, 28, and 36 DEGs were related to carbohydrate metabolism under JA, SA, and COR treatments. Cellulose, a kind of polysaccharide is the central component in the plant secondary wall. Cellulose synthesis is a highly regulated process involving multi protein complexes including the cellulose synthase (CESA) complex from the glycosyltransferase GT-2 family (Decker et al., 2012). In our analysis, genes related with cellulose biosynthesis were up-regulated in all three compound treated samples (Supplementary Table 3). The gene GALE (LOC101266745) encoding UDP-glucose 4-epimerase (UGE) which catalyzes the conversion of UPD-Galactose to UDP-Glucose was 2-fold up-regulated by JA, whereas genes encoding cellulose degradation enzymes such as LOC101247302, CEl7 (LOC543583), and CELl8 (LOC543583) were all down-regulated by JA and SA (Supplementary Tables 3, 4). This result indicated that SA and JA treatments induced cellulose synthesis to prevent TYLCV invasion. In addition, many genes involved in sugar metabolism that were differentially regulated by the three compounds. For example, Wiv-1 (LOC543502) encoding beta-fructofuranosidase, TPS1 (LOC100135703) encoding trehalose 6-phosphate synthase were up-regulated in all three compound treated samples (Supplementary Table 3). LOC101257623 that encodes raffinose synthase, was up-regulated under JA and SA treatments. Specifically, for COR treatment, XET2 (xyloglucan endotransglycosylase 2), XET4 (xyloglucan endotransglycosylase 4), XTH7 (xyloglucan endotransglucosylase-hydrolase 7), and XTH1 (endo-xyloglucan transferase 1), all of which are involved in the plant cell wall development (Decker et al., 2012) were up-regulated after TYLCV invasion (Supplementary Table 3). Taken together, all three treatments activated plant defense against TYLCV through regulating cell wall related physiological processes.

### Impact on plant hormone signal transduction pathways

Plant hormones play key regulatory roles during the activation of specific defense signaling pathways in response to pathogen attack. Pathogens can also manipulate plant hormone signaling pathways to evade host defenses. The exogenous application of SA enhanced the resistance of plants infected with TYLCV by inducing the expression of PR (pathogenesis related) genes (Li T. et al., 2019). Our data also showed that *SlPR1b1*, which serves as a readout for SA signaling (Li et al., 2017) was strongly up-regulated by SA and down-regulated by JA after TVLCV invasion (Supplementary Tables 5, 6), thereby suggesting that SA and JA might induce tomato resistance against TYLCV through different mechanisms. Brassinosteroids (BRs) can enhance plants resistance by inducing ROS generation in *Nicotiana benthamiana* (Deng et al., 2016). As the negative regulator in BR signaling pathway, BKI1 (BRI1 kinase inhibitor 1) prevents the activation of BR signaling transduction by interaction with BRI1 (brassinosteroid insensitive 1), the plasma membrane-localized receptor of BRs (Wang and Chory, 2006). In our study, CYCD3 (cyclin D3), the gene encoding a D-type plant cyclin protein, involved in the BR pathway, was down-regulated in all three treatments (Supplementary Table 6), whereas BKI1 (LOC101262109) was strongly up-regulated only by SA treatment (Supplementary Table 5).

Auxin is essential for plant growth and orchestrates many developmental processes (Dharmasiri and Estelle, 2004). In our study, many genes encoding auxin-responsive proteins (IAA), such as IAA2, IAA3, IAA19, IAA21, and LOC101055550, were all down-regulated in the presence of JA, SA and COR (Supplementary Table 6). Meanwhile, SAUR (small auxin-up RNA) genes (LOC101252930, LOC101247499, and LOC101249503), which are involved in tryptophan metabolism, were also down-regulated by JA, SA and COR (Supplementary Table 6). These results indicate that the three compound treatments perturbed auxin and tryptophan signaling, as these negatively regulate defenses against TYLCV invasion.

### Secondary metabolism regulated by salicylic acid, jasmonate acid, and coronatine

In tomato plants infected with TYLCV, many secondary metabolites play a key role in response to the virus. 46 DEGs regulated by JA were involved in the Phenylalanine pathway. Among the 46 DEGs involved in phenolic metabolism, genes encoding phenylalanine ammonia-lyase (PAL), 4-coumarate: CoA ligase (4CL), shikimate O-hydroxy cinnamoyl transferase (HCT) and caffeoyl-CoA O-methyltransferase were induced by JA (Supplementary Table 7). Interestingly, the expression of 4CL was up-regulated under all three treatments with the highest induction observed in JA treated samples (Supplementary Table 7). The genes involved in flavonoid biosynthesis such as F3H (flavanone-3-dioxygenase), F3′5′H (flavonoid-3′,5′-hydroxylase), CHI1 (chalcone–flavonone isomerase 1), CHS1 (chalcone synthase 1) and CHS2 (chalcone synthase 2) were all down-regulated in the presence of the compounds (Supplementary Table 8) with the most significant reduction in transcript abundance observed under SA and COR treatments.

Some amino acids, such as serine, proline, and leucine, have been showed as precursors for the synthesis of secondary metabolites, which play pivotal roles in response to pathogens (Hildebrandt et al., 2015). In this study, the expression of three genes (LOC101259064, LOC101261704, and LOC101258774) involved in tyrosine metabolism were up-regulated by JA (Supplementary Table 7). Three genes involved in the linoleic acid metabolism pathway, two genes encoding lipoxygenase (LoxC and LOC101262081) were also up-regulated by JA and down-regulated in the presence of SA and COR (Supplementary Tables 7, 8). The expression of Lox genes under the exogenous application of JA was induced but repressed by the exogenous application of SA and COR. Taken together, the expression of genes involved in phenylpropanoid metabolism and amino acids metabolism was affected by the exogenous application of JA.

### The tomato response to tomato yellow leaf curl virus infection in *SlNPR1* and *SlCOI1* silenced plants

**FIGURE 6:**
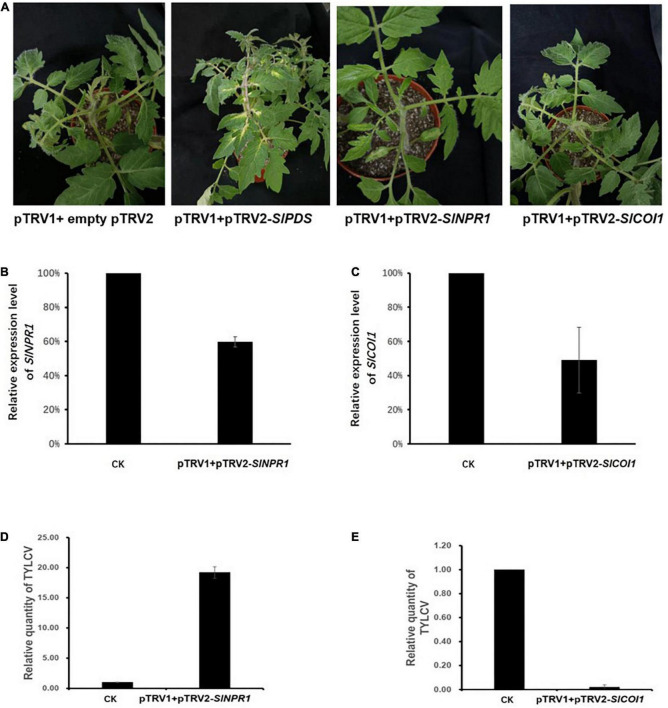
Tomato yellow leaf curl virus (TYLCV) levels were affected by silencing essential genes in the salicylic acid (SA) and jasmonate acid (JA) signaling pathways. **(A)** The first picture (from left to right) shows the negative control plant carrying pTRV1 + empty pTRV2. The second picture shows the positive control plant carrying pTRV1 + pTRV2-*SlPDS.* The bleached areas in leaves were visible 2 weeks after Agro-inoculation. The third picture shows plants Agro-infiltrated with pTRV1 + pTRV2-*SlNPR1* while the forth picture depicts plants Agro-infiltrated with pTRV1 + pTRV2*-SlCOI1.* **(B)** The relatively expression levels of *SlNPR1* in pTRV1 + pTRV2-*SlNPR1* Agro-inoculated plants. **(C)** The relatively expression levels of *SlCOI1* in pTRV1 + pTRV2-*SlCOI1* Agro-inoculated plants. The *SlNPR1* or *SlCOI1* expression profile in the pTRV1 + empty pTRV2 inoculation plants (CK) was set to 1 and served as the baseline. Shown is representative data from three independent biological replicates. **(D,E)** The relative levels of TYLCV in pTRV1pTRV2-*SlNPR1* plants and pTRV1 + pTRV2-*SlCOI1* plants 7 days post inoculation of TYLCV. CK represents the baseline TYLCV levels in plants with pTRV1 + empty pTRV2 agroinfiltration. The mean and SEM are derived from pooled data corresponding to three independent biological replicates.

To further investigate the role of JA, COR, and SA induced defense against TYLCV invasion, we silenced *SlNPR1*, an essential contributor to SA signaling, and *SlCOI1*, a key component of the JA singling pathway. We proceeded to detect TYLCV levels in the silenced plants, even though our data analysis showed that *SlCOI1* expression levels remained largely unaffected and *SlNPR1* expression was only weakly up-regulated following compound treatments. The positive control (plant with pTRV1 + pTRV2-*SlPDS)* showed bleached areas in leaves after 2 weeks of agroinfiltration (Figure 6A). We measured the relative expression level of *SlNPR1* and *SlCOI1* after silencing the two genes. Compared to the control plants, the expression level of *SlNPR1 and SlCOI1* in the silenced plants decreased by approximately 40% and 50%, respectively (Figures 6B,C). Further, we detected TYLCV accumulation 1 week after inoculation of the viral clone in the silenced plants. Our results showed that the amount of TYLCV accumulation in *SlNPR1* silencing plants was approximately 19 times greater than in the control plants (Figure 6D). This indicated to us that *SlNPR1* is required for SA mediated defense activation in response to TYLCV infection. Surprisingly, a dramatic reduction in the level of TYLCV accumulation was not observed in the *SlCOI1* silenced plants (Figure 6E). Based on previous reports, *coi1* mutant plants exhibited enhanced resistance and higher SA accumulation at early stages of bacterial infection (Kloek et al., 2001). We therefore speculate that partial *SlCOI1* silencing may have led to hyper-activated SA signaling thereby resulting in a reduction in TYLCV accumulation.

### Validation with quantitative real-time RT-PCR of RNA sequencing results

**FIGURE 7:**
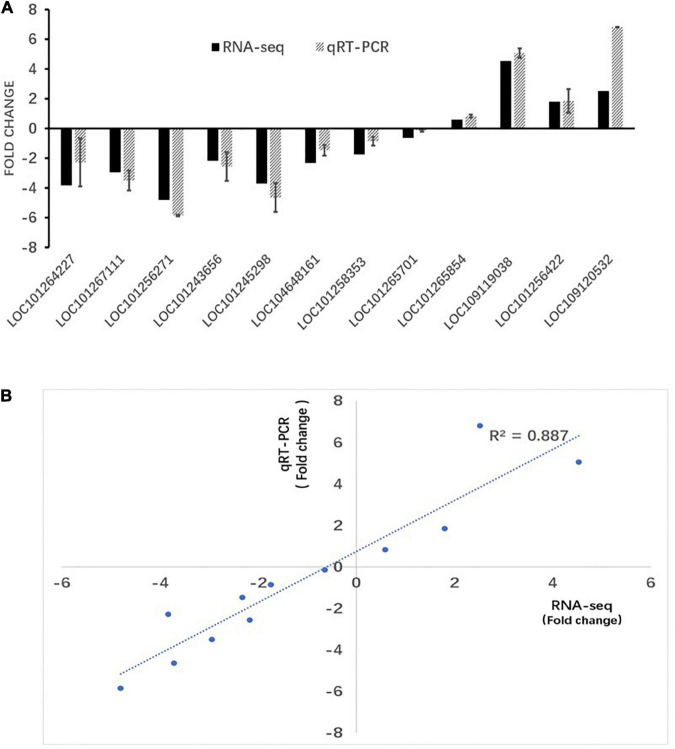
RT-PCR verification of RNA-seq results. **(A)** RT-qPCR and RNA-seq comparison between 12 randomly selected differentially expressed genes (DEGs). The RT-qPCR values represent mean ± SEM from three biological replicates. **(B)** Regression analysis of gene expression ratios compared by RT-qPCR and RNA-seq analysis.

To validate the reliability of the RNA-seq data, 12 differentially expressed genes were randomly selected for qRT-PCR validation (Figure 7). These genes included LOC101264227, LOC101267111, LOC101256271, LOC101 243656, LOC101245298, LOC104648161, LOC101258353, LOC101265701, LOC101265854, LOC109119038, LOC1012 56422, and LOC109120532. Our results showed a linear correlation (R^2^ > 0.887) between the fold change value from the RNA-seq and qRT-PCR experiments, which suggested that the RNA-seq data was reliable.

## Discussion

Tomato yellow leaf curl virus is a devastating pathogen that has caused significant reduction in yield worldwide. The initial response to pathogen ingress includes the production of ROS (Lamb and Dixon, 1997). Previous reports suggested that one of the main sources of ROS during the oxidative burst is peroxidase-derived H_2_O_2_ (O’Brien et al., 2012). We found that JA, SA, and COR treatments could reduce TYLCV accumulation (Figure 1), and that all three treatments resulted in H_2_O_2_ production at the early stage of infection (Figure 2). It is known that H_2_O_2_ production is caused by the partial influx of Ca^2+^ (O’Brien et al., 2012). As expected, the genes (LOC101055527 and LOC101260391) encoding CDPK (calcium-dependent protein kinase) and (LOC109120532) encoding calmodulin (CAM) were highly up-regulated by JA in response to TYLCV infection and weakly upregulated by SA and COR. This suggests that the production of Ca^2+^ which is dependent on CDPK activation contributes to ROS production. In addition, several genes involved in peroxidase metabolism were upregulated only in the presence of JA. These included FAR (fatty acyl-CoA reductase) and XDH (xanthine dehydrogenase/oxidase). These results might help explain why JA treatment induced higher level of ROS production than either SA or COR.

The cell wall provides rigidity to the cell and serves as a passive barrier that limits the access of pathogens to the cells. It is therefore not surprising that our data analysis showed that many genes in cell wall category were enriched in JA, SA and COR treated samples. Some common genes were shared between the three treatments. These included genes related to the cell wall biosynthesis such as Wiv-1 (LOC543502), TPS1, GALE, and UGE (Rosti et al., 2007). We identified 369 DEGs in common under the three treatments (Figure 5A and Supplementary Tables 1, 2). Interestingly many genes involved in auxin signaling was down-regulated in the presence of the compounds (Supplementary Table 6). Considering the trade-off between plant growth and plant defense, it is not surprising that genes related to auxin signal pathways were suppressed. Viral accumulation shifts the nutrient allocation from plant growth to defense. Thus, the activation of plant defenses affects plant growth and development, resulting in a reduction in overall yield.

In plants, many secondary metabolites with roles in plant defense are derived from the different branches of the phenylpropanoid pathway (Naoumkina et al., 2010; Piasecka et al., 2015). In this study, we identified many genes related to the phenylpropanoid pathway that were induced in the presence of all three compounds. For instance PAL and 4CL, two genes in the PAL pathway were up-regulated, while genes involved in flavonoid biosynthesis such as F3H, F3′5′H, were all down-regulated. These results indicated that while the phenylpropanoid pathway might be induced upon the three treatments, there are multiple downstream branches which might be involved in many intermediated products that function as phytoalexins to defend against the virus. The C2 protein, which is encoded by TYLCV, has been showed to suppress JA responses by interacting with plant ubiquitin, which results in the degradation of JAZ1 protein, thereby leading to a reduction in JA mediated defenses (Li P. et al., 2019). βC1 protein encoded by the betasatellite of tomato yellow leaf curl China virus (TYLCCNB-βC1), suppresses JA dependent terpene biosynthesis in plants (Li et al., 2014). Our analysis showed that COR, as a mimic of JA, induced many genes that overlapped with JA treatment during TYLCV infection. Our results are consistent with many previous reports that showed that COR and JA share similar functions such as the activation of JA responses and the elicitation of secondary metabolism (Thilmony et al., 2006). The identification of secondary compounds is an emergent area of research and will contribute to advancements in agricultural research through the development of effective plant defense strategies.

We report that *SlNPR1* silenced plant accumulated more TYLCV than wild type plants after Agro-inoculation of TYLCV. However, in comparison with wild type plants, in *SlCOI1* silenced plants, there was a diminution in TYLCV levels. We speculate that SA signaling pathway might by hyper-activated when *SlCOI1* is partly silenced, thereby resulting in a reduction in TYLCV levels. To date, several key regulatory proteins involved in SA-JA crosstalk have been identified in *Arabidopsis*. NPR1, the major positive regulator of SA signaling, is a possible JA-SA crosstalk mediator (Dong, 2004) and requires nuclear localization for efficient function (Spoel et al., 2003). Interestingly *coi1* mutant plants display enhanced resistance against biotrophic pathogens, and accumulated a greater amount of SA at early stages of bacterial infection (Kloek et al., 2001). Based on our transcriptomics data, *SlPR1b* expression was inhibited by JA treatment, which indicated that SA signaling pathway was suppressed by JA treatment. Further, considering there was only 50% reduction in *SlCOI1* expression through the VIGS system, JA signaling would still be partially activated upon TYLCV invasion. To conclude, in *SICOI1* silenced plants, low levels of JA accumulation combined with a hyper-activated SA response may lead to a robust immune response against TYLCV accumulation. In future, the generation of null *SlCOI1* lines would further aid in determining the function of SlCOI1 during TYLCV tomato interactions.

## Conclusion

Tomato yellow leaf curl virus infection causes substantial losses in tomato production worldwide. Based on our findings, SA, JA, and COR inhibit TYLCV accumulation at the early stage of TYLCV infection. We carried out RNA-Seq to identify DEGs induced under three different treatments. In addition, we silenced *SlNPR1* and *SlCOI1* in tomato to investigate the role of SA and JA induced systemic resistance in defense against TYLCV infection. Our findings will serve as a valuable resource for understanding the mechanistic details underlying TYLCV tomato interactions.

## Data availability statement

The datasets presented in this study can be found in online repositories. The names of the repository/repositories and accession number(s) can be found in the article/Supplementary material.

## Author contributions

PW and SS: conceptualization. PW, SS, and KL: formal analysis. SS and XG: funding acquisition and supervision. PW, KL, RP, NL, and BH: methodology. SS, AJA, and XG: writing—original draft. AJA and XG: writing—review and editing. All authors contributed to the article and approved the submitted version.

## Footnotes

1
https://www.ncbi.nlm.nih.gov/assembly/GCF_000188115.4

2
http://geneontology.org/

3
https://www.genome.jp/kegg/

4
https://www.ncbi.nlm.nih.gov/geo/

5
https://www.ncbi.nlm.nih.gov/assembly/GCF_000188115.3/
