# Supplementary material for: Physiological and transcriptomic analyses revealed gene networks involved in heightened resistance against tomato yellow leaf curl virus infection in salicylic acid and jasmonic acid treated tomato plants
Source: Front Microbiol. 2022 Sep 14;13:970139. doi: 10.3389/fmicb.2022.970139 (PMC9515787; doi:10.3389/fmicb.2022.970139)
Supplement: Supplementary file 1 [file Table_1.DOCX]

**Table S1. The shared DEGs up-regulates by JA、SA and COR treatment**

| **Gene ID** | **Gene Symbol** | **log2 (COR_T / MOCK)** | **log2 (JA_T / MOCK)** | **log2 (SA_T / MOCK)** |
| --- | --- | --- | --- | --- |
| 100134896 | 5PT1 | 2.217916724 | 2.466580148 | 4.663195488 |
| 100134911 | JAZ2 | 1.054627079 | 1.287634917 | 2.125127387 |
| 100191111 | LOC100191111 | 1.462219436 | 2.287761492 | 4.489805268 |
| 100316872 | LOC100316872 | 1.987927168 | 2.510961919 | 3.978805778 |
| 100736441 | LOC100736441 | 1.037389007 | 1.68778706 | 1.499285676 |
| 100736460 | TCP23 | 1.157336538 | 1.032289538 | 1.327852929 |
| 101055527 | LOC101055527 | 1.047048573 | 2.145212048 | 2.24610849 |
| 101055566 | TIP2-2 | 1.73396047 | 1.390958146 | 1.399447386 |
| 101243631 | LOC101243631 | 1.927654775 | 1.866130594 | 2.664982226 |
| 101243853 | LOC101243853 | 2.61272154 | 4.379546692 | 3.561414855 |
| 101243873 | LOC101243873 | 2.889612604 | 1.514573173 | 1.934112064 |
| 101244246 | LOC101244246 | 1.097322073 | 1.187989717 | 2.417752153 |
| 101244324 | LOC101244324 | 1.656441889 | 2.231198319 | 1.445625819 |
| 101244398 | LOC101244398 | 1.393663848 | 2.773279294 | 2.762202886 |
| 101244399 | LOC101244399 | 1.187331278 | 3.204530047 | 4.575332672 |
| 101244780 | LOC101244780 | 1.505395243 | 1.374445782 | 1.664815808 |
| 101244788 | LOC101244788 | 1.537965021 | 1.182203331 | 1.132450296 |
| 101244915 | LOC101244915 | 1.515038483 | 1.639754974 | 1.770999507 |
| 101244953 | PHO1-3 | 2.198328716 | 1.191695624 | 2.458050388 |
| 101245027 | LOC101245027 | 1.611434712 | 3.019899557 | 4.256339753 |
| 101245143 | LOC101245143 | 1.164331605 | 1.971915254 | 2.982851718 |
| 101245220 | CER6 | 1.224362992 | 2.07127302 | 2.040248586 |
| 101245282 | LOC101245282 | 1.430783828 | 1.716744201 | 1.8183461 |
| 101245301 | SN2 | 2.310194041 | 1.885903621 | 3.072748374 |
| 101246079 | LOC101246079 | 1.199308808 | 1.013296823 | 2.772589504 |
| 101246440 | LOC101246440 | 2.357216845 | 1.373217324 | 2.612183969 |
| 101246511 | LOC101246511 | 2.204013892 | 1.347270943 | 1.332843872 |
| 101246543 | LOC101246543 | 1.233000521 | 1.39358439 | 1.255472861 |
| 101246620 | LOC101246620 | 1.328269622 | 1.6732974 | 2.820909939 |
| 101246714 | LOC101246714 | 4.137503524 | 3.786596362 | 5.510961919 |
| 101246932 | LOC101246932 | 1.189824559 | 1.754887502 | 2.764871591 |
| 101247047 | LOC101247047 | 1.597667661 | 1.797534738 | 2.401499284 |
| 101247242 | LOC101247242 | 2.123631349 | 2.953706348 | 3.044854236 |
| 101247504 | LOC101247504 | 1.05621601 | 1.616463644 | 1.701174475 |
| 101247747 | PIP2-1 | 1.284205789 | 1.044744401 | 1.040874508 |
| 101247953 | LOC101247953 | 2.831602468 | 3.623168505 | 6.117356951 |
| 101248212 | LOC101248212 | 1.759865996 | 1.50589093 | 1.478540442 |
| 101248276 | LOC101248276 | 2.488016945 | 2.68993206 | 2.433631366 |
| 101248628 | LOC101248628 | 3.409390936 | 3.189824559 | 3.14974712 |
| 101248631 | LOC101248631 | 1.258461676 | 1.656221184 | 2.02713613 |
| 101248884 | TRM16/32a | 1.044394119 | 1.609794354 | 2.63420602 |
| 101248906 | LOC101248906 | 1.234465254 | 1.08912589 | 1.815414692 |
| 101248957 | LOC101248957 | 1.39665897 | 1.516398214 | 2.004558282 |
| 101249225 | LOC101249225 | 1.037121131 | 1.640292057 | 1.968639273 |
| 101249567 | LOC101249567 | 1.093109404 | 1.457681837 | 1.742724863 |
| 101249694 | LOC101249694 | 1.350411782 | 1.12116569 | 1.360631624 |
| 101250073 | LOC101250073 | 2.580713028 | 1.03355256 | 2.021061616 |
| 101250102 | LOC101250102 | 1.096844078 | 1.443824135 | 1.454166079 |
| 101250146 | LOC101250146 | 1.010530667 | 1.054447784 | 1.145647598 |
| 101250147 | LOC101250147 | 1.476438044 | 1.346802764 | 1.960253821 |
| 101250247 | LOC101250247 | 1.376982737 | 3.315457484 | 3.915144022 |
| 101250284 | LOC101250284 | 1.08865762 | 1.284881108 | 1.212196654 |
| 101250295 | LOC101250295 | 1.938599455 | 1.852442812 | 2.247927513 |
| 101250453 | LOC101250453 | 1.465534685 | 1.731723509 | 2.49102229 |
| 101250696 | LOC101250696 | 1.043569015 | 1.524744978 | 1.302746682 |
| 101250760 | LOC101250760 | 1.384859351 | 1.741552864 | 1.603224707 |
| 101250924 | LOC101250924 | 3.308752706 | 2.169925001 | 4.42931163 |
| 101251005 | LOC101251005 | 2.321928095 | 2.600729817 | 2.933074527 |
| 101251133 | LOC101251133 | 1.963058422 | 1.24991685 | 1.208430747 |
| 101251368 | LOC101251368 | 2.021149835 | 2.801254723 | 3.916194423 |
| 101251402 | LOC101251402 | 1.292781749 | 1.252063288 | 2.971020933 |
| 101251439 | LOC101251439 | 1.169925001 | 2.19061486 | 1.632268215 |
| 101251681 | LOC101251681 | 2.280107919 | 1.885136043 | 1.908852112 |
| 101251721 | LOC101251721 | 1.596397396 | 1.501937469 | 1.075028661 |
| 101251769 | LOC101251769 | 1.785610802 | 2.245938412 | 1.967535605 |
| 101251885 | LOC101251885 | 6.754887502 | 6.845490051 | 5.906890596 |
| 101251972 | LOC101251972 | 1.212993723 | 2.025995209 | 3.605580532 |
| 101252072 | LOC101252072 | 2.191365526 | 2.890425994 | 3.772963792 |
| 101252329 | LOC101252329 | 1.925999419 | 1.666756592 | 2.270528942 |
| 101252447 | LOC101252447 | 1.470791481 | 2.943534078 | 1.197537233 |
| 101252571 | LOC101252571 | 1.811842313 | 3.165924458 | 3.154360714 |
| 101252644 | LOC101252644 | 1.54299023 | 1.15083916 | 1.042041771 |
| 101252712 | LOC101252712 | 1.17555687 | 1.832923013 | 1.361263267 |
| 101252838 | LOC101252838 | 1.160108143 | 1.301463992 | 1.817959477 |
| 101252940 | LOC101252940 | 2.131626957 | 2.770012495 | 1.500428991 |
| 101253053 | LOC101253053 | 1.83720485 | 1.856767116 | 1.650876617 |
| 101253328 | LOC101253328 | 1.264827688 | 1.46529227 | 2.157912484 |
| 101253448 | LOC101253448 | 1.295219696 | 1.273623041 | 1.601867213 |
| 101253579 | LOC101253579 | 2.321928095 | 3.674599713 | 2.714597781 |
| 101253604 | LOC101253604 | 1.07287888 | 1.502939986 | 1.799860386 |
| 101253738 | LOC101253738 | 2.402098444 | 2.807354922 | 3.123382416 |
| 101253859 | LOC101253859 | 2.816288047 | 4.410128699 | 2.134649527 |
| 101254097 | LOC101254097 | 1.058736439 | 1.618315482 | 1.982946961 |
| 101254202 | LOC101254202 | 1.72038271 | 3.480511337 | 3.399496648 |
| 101254611 | LOC101254611 | 1.32334877 | 1.326185929 | 2.031631609 |
| 101254713 | LOC101254713 | 1.443799366 | 1.334411507 | 2.414600584 |
| 101254719 | LOC101254719 | 1.192645078 | 1.175367087 | 1.655196038 |
| 101255038 | LOC101255038 | 3.044394119 | 4.554588852 | 4.459431619 |
| 101255309 | LOC101255309 | 1.420938101 | 1.58758797 | 2.224080771 |
| 101255316 | LOC101255316 | 3.247927513 | 3.554588852 | 4.781359714 |
| 101255412 | LOC101255412 | 2.790076931 | 2.025535092 | 2.356993362 |
| 101255631 | LOC101255631 | 1.609837169 | 1.476438044 | 1.95864097 |
| 101255659 | LOC101255659 | 1.810945206 | 1.9224634 | 1.203091865 |
| 101256182 | LOC101256182 | 2.831400396 | 2.967819594 | 3.474779583 |
| 101256205 | LOC101256205 | 2.985303489 | 2.851242566 | 2.744742945 |
| 101256373 | LOC101256373 | 1.798457446 | 2.3959652 | 2.239251072 |
| 101256578 | LOC101256578 | 1.138130919 | 1.272780446 | 2.565270653 |
| 101257137 | LOC101257137 | 2.325027334 | 1.248743748 | 1.853158612 |
| 101257172 | TRM18 | 1.010416616 | 1.468904371 | 2.061400545 |
| 101257456 | LOC101257456 | 1.113890967 | 2.045642666 | 2.007708095 |
| 101257475 | CNGC5 | 1.062541466 | 1.296132094 | 2.139162748 |
| 101257483 | LOC101257483 | 2.495410916 | 1.67556505 | 1.389770741 |
| 101257981 | LOC101257981 | 1.0691397 | 2.294386825 | 2.119507094 |
| 101258062 | LOC101258062 | 1.111343587 | 1.901258061 | 1.949688736 |
| 101258128 | LOC101258128 | 1.167294745 | 1.6617197 | 2.873393684 |
| 101258203 | LOC101258203 | 1.296531318 | 1.133978305 | 1.800422208 |
| 101258372 | LOC101258372 | 1.64479575 | 2.04965347 | 1.075527057 |
| 101258514 | LOC101258514 | 1.205881348 | 1.407837145 | 2.40109077 |
| 101258830 | LOC101258830 | 2.532495081 | 2.402098444 | 2.822001698 |
| 101258998 | MCA2 | 2.456715531 | 1.569960337 | 1.681177816 |
| 101259165 | LOC101259165 | 1.312857104 | 1.616805384 | 1.916993735 |
| 101259234 | LOC101259234 | 1.361379246 | 1.119865647 | 1.300341233 |
| 101259357 | LOC101259357 | 1.218349197 | 2.288922554 | 1.09326998 |
| 101259430 | LOC101259430 | 1.312277925 | 2.625790005 | 1.657894023 |
| 101259605 | LOC101259605 | 1.236440196 | 1.505983711 | 2.287259078 |
| 101259925 | LOC101259925 | 1.190683562 | 1.150434094 | 2.280210409 |
| 101259970 | SAP8 | 1.269389901 | 2.714336508 | 2.695285916 |
| 101259986 | LOC101259986 | 1.030287476 | 1.820530311 | 1.540723479 |
| 101260016 | LOC101260016 | 1.229133999 | 1.6617197 | 2.040806216 |
| 101260225 | LOC101260225 | 1.99841723 | 1.356934545 | 1.67688499 |
| 101260459 | LOC101260459 | 2.086678522 | 1.360450137 | 1.439037093 |
| 101260610 | LOC101260610 | 1.073500022 | 3.084240521 | 2.95699194 |
| 101260643 | ABCG39 | 3.247927513 | 5.491853096 | 6.95419631 |
| 101260894 | LOC101260894 | 1.254286094 | 2.743517726 | 2.170380973 |
| 101261020 | LOC101261020 | 2.867309326 | 2.603883363 | 2.338967998 |
| 101261024 | LOC101261024 | 1.626541604 | 1.935294311 | 2.326981323 |
| 101261157 | LOC101261157 | 1.075707251 | 1.378511623 | 2.133751389 |
| 101261239 | STP11 | 1.529365909 | 1.670738308 | 2.946625524 |
| 101261573 | LOC101261573 | 1.065906181 | 1.394456622 | 1.986655874 |
| 101261743 | LOC101261743 | 2.337235348 | 2.275007047 | 3.056583528 |
| 101262036 | LOC101262036 | 1.268841344 | 1.351711564 | 1.504061417 |
| 101262285 | LOC101262285 | 1.104108403 | 3.572642229 | 5.343842871 |
| 101262373 | LOC101262373 | 1.775627215 | 1.569042946 | 1.858185705 |
| 101262489 | LOC101262489 | 2.316145742 | 2.722466024 | 3.765534746 |
| 101262589 | LOC101262589 | 1.062664035 | 1.097736802 | 1.663674477 |
| 101262709 | LOC101262709 | 1.453956489 | 2.073248982 | 1.163230349 |
| 101262853 | ACAT4 | 1.246994395 | 3.381983979 | 2.14689856 |
| 101263024 | LOC101263024 | 1.142444265 | 1.083293663 | 1.353163474 |
| 101263195 | LOC101263195 | 2.190736407 | 1.86332856 | 1.735855402 |
| 101263269 | LOC101263269 | 2.732330644 | 3.803850871 | 2.578596991 |
| 101263388 | LOC101263388 | 7.392317423 | 6.686500527 | 6.475733431 |
| 101263487 | LOC101263487 | 1.658963082 | 2.314314911 | 2.658963082 |
| 101263662 | LOC101263662 | 1.007159792 | 1.428433093 | 2.07175584 |
| 101263761 | LOC101263761 | 1.330824495 | 1.559958495 | 1.264273302 |
| 101263824 | LOC101263824 | 2.845490051 | 5.461479447 | 3.87036472 |
| 101264257 | LOC101264257 | 3.257797757 | 1.771058793 | 4.278954409 |
| 101264419 | LOC101264419 | 1.383591115 | 1.67649605 | 3.088944428 |
| 101264719 | RAV2 | 1.031686393 | 1.392120562 | 1.602765323 |
| 101264738 | LOC101264738 | 1.0128448 | 1.998239542 | 1.739777866 |
| 101265127 | LOC101265127 | 1.721813369 | 1.14822993 | 1.843803893 |
| 101265204 | LOC101265204 | 1.556393349 | 1.640457613 | 1.934904972 |
| 101265282 | AMT1-1 | 1.062572471 | 2.291946252 | 2.697288007 |
| 101265373 | LOC101265373 | 1.853067896 | 1.167665494 | 1.373814837 |
| 101265482 | LOC101265482 | 1.053315815 | 1.296586966 | 1.080858274 |
| 101265528 | LOC101265528 | 1.166192283 | 1.551015169 | 1.355094959 |
| 101265539 | LOC101265539 | 2.321928095 | 3.502500341 | 5.00873985 |
| 101265550 | LOC101265550 | 1.335326934 | 1.397741879 | 1.367439119 |
| 101265571 | LOC101265571 | 1.475511358 | 2.067563284 | 1.565062943 |
| 101265598 | LOC101265598 | 1.229375868 | 2.247092862 | 1.693661011 |
| 101265652 | LOC101265652 | 2.465974465 | 4.756222713 | 3.981852653 |
| 101265775 | LOC101265775 | 1.717221659 | 2.823933995 | 2.705816896 |
| 101265906 | LOC101265906 | 2.257496222 | 1.741931847 | 1.655088587 |
| 101265918 | LOC101265918 | 2.545649577 | 2.455194626 | 2.340424439 |
| 101265959 | LOC101265959 | 2.702903758 | 1.49683159 | 1.975752454 |
| 101266119 | LOC101266119 | 1.424026283 | 3.497186541 | 3.767301673 |
| 101266208 | LOC101266208 | 1.454378391 | 2.151309323 | 1.925269125 |
| 101266266 | LOC101266266 | 2.960772596 | 4.333337734 | 1.64558886 |
| 101266657 | LOC101266657 | 1.467868456 | 2.664189192 | 2.095652239 |
| 101266718 | LOC101266718 | 1.202579168 | 1.923708214 | 2.708780626 |
| 101266750 | LOC101266750 | 1.042774024 | 2.057289803 | 1.645991934 |
| 101266868 | LOC101266868 | 2.624626718 | 4.248192058 | 1.58580006 |
| 101266892 | LOC101266892 | 1.567989422 | 1.306661338 | 2.457128833 |
| 101266963 | LOC101266963 | 1.263776342 | 2.111361109 | 1.241350579 |
| 101266965 | LOC101266965 | 1.34614549 | 1.349698928 | 1.682587386 |
| 101267150 | ABCG1 | 1.11478489 | 1.326519808 | 2.201025166 |
| 101267164 | LOC101267164 | 1.029443778 | 1.805228621 | 1.619255678 |
| 101267223 | LOC101267223 | 4.345774837 | 4.36923381 | 4.087462841 |
| 101267279 | LOC101267279 | 1.813903708 | 1.348032694 | 2.326820664 |
| 101267565 | LOC101267565 | 1.165202591 | 1.443918619 | 1.967819594 |
| 101267713 | LOC101267713 | 2.251398062 | 2.145059744 | 2.127333575 |
| 101267799 | LOC101267799 | 4.117695043 | 1.8372705 | 2.111031312 |
| 101267893 | CYP77A19 | 1.230976724 | 1.191938625 | 1.58872442 |
| 101268306 | LOC101268306 | 1.065963262 | 1.272145457 | 2.227805918 |
| 101268316 | LOC101268316 | 2.434849607 | 2.162814612 | 1.369645596 |
| 101268692 | LOC101268692 | 1.584962501 | 4.090341167 | 3.87780483 |
| 101268830 | LOC101268830 | 2.231096031 | 2.949999537 | 2.57996825 |
| 104644303 | LOC104644303 | 1.76650987 | 3.549286769 | 4.639870842 |
| 104645408 | LOC104645408 | 1.503962779 | 2.090386236 | 1.771375625 |
| 104645747 | LOC104645747 | 1.014950341 | 2.095796584 | 1.964034503 |
| 104645857 | LOC104645857 | 4.087462841 | 3.906890596 | 6.392317423 |
| 104646741 | LOC104646741 | 1.075717054 | 1.389042291 | 1.436570661 |
| 104648557 | LOC104648557 | 1.593121024 | 1.319198302 | 1.414355535 |
| 109120085 | LOC109120085 | 1.444011675 | 1.359314511 | 1.672897859 |
| 112940036 | LOC112940036 | 3.963474124 | 2.232660757 | 3.95419631 |
| 112940330 | LOC112940330 | 6.569855608 | 9.556506055 | 9.25502857 |
| 112941696 | LOC112941696 | 3.502500341 | 4.874469118 | 4.64385619 |
| 543502 | Wiv-1 | 1.840521786 | 2.713286598 | 3.568088682 |
| 543511 | LOC543511 | 1.242714771 | 2.755698528 | 3.905745615 |
| 543522 | ME2 | 1.025772931 | 1.190253793 | 1.445605613 |
| 543530 | ProT1 | 1.354572858 | 1.058032121 | 1.729409374 |
| 543619 | XET2 | 2.752416529 | 3.287110417 | 3.832973545 |
| 543758 | LOC543758 | 1.067214272 | 1 | 2.197247637 |
| 543824 | AOX1a | 1.192098499 | 1.764248349 | 2.861895228 |
| 544028 | ACS1A | 2.357552005 | 4.238404739 | 4.214319121 |
| 544052 | ACO1 | 1.897430266 | 4.223907062 | 4.612976877 |
| 544084 | CEVI-1 | 3.46317402 | 1.192645078 | 2.21649182 |
| 544149 | CHI3 | 1.823317632 | 1.458205358 | 3.100359407 |
| 544254 | THT1-3 | 1.681366985 | 2.953060777 | 3.300659478 |
| 544271 | ARG2 | 1.244360898 | 2.856793103 | 1.356653932 |
| 606711 | AOS | 1.464281018 | 3.222392421 | 2.955918932 |
| 778256 | AADC2 | 2.627273306 | 4.006426269 | 3.025535092 |
| 778266 | LOC778266 | 5 | 4.36923381 | 4.700439718 |
| 778318 | LOC778318 | 1.283938509 | 1.206586718 | 1.253835469 |

**Table S2. The shared DEGs down-regulates by JA、SA and COR treatment**

| **Gene ID** | **Gene Symbol** | **log2 (COR_T / MOCK)** | **log2 (JA_T / MOCK)** | **log2 (SA_T / MOCK)** |
| --- | --- | --- | --- | --- |
| 100301942 | Style2.1 | -1.949496767 | -3.00152747 | -2.286738666 |
| 100736480 | IAA21 | -2.079727192 | -3.784271309 | -3.409875794 |
| 101055542 | IAA2 | -1.216575095 | -1.150384815 | -1.196397213 |
| 101055549 | IAA19 | -1.813016257 | -1.44164923 | -1.592515895 |
| 101055550 | LOC101055550 | -1.820144363 | -2.668141269 | -3.05300062 |
| 101244240 | LOC101244240 | -1.270601514 | -1.270601514 | -1.111611991 |
| 101244813 | LOC101244813 | -1.307822328 | -2.53542304 | -2.730679331 |
| 101244876 | LOC101244876 | -1.142703533 | -1.412495305 | -1.249359469 |
| 101244897 | LOC101244897 | -1.329705445 | -1.45169597 | -1.924448967 |
| 101245083 | LOC101245083 | -1.226609598 | -1.579918107 | -1.520722315 |
| 101245346 | LOC101245346 | -1.861923865 | -1.53542304 | -2.839203788 |
| 101245452 | LOC101245452 | -2.533179959 | -2.233619677 | -4.911691582 |
| 101245569 | LOC101245569 | -2.231099117 | -1.62188907 | -3.59018021 |
| 101245724 | LOC101245724 | -1.968521769 | -1.756545963 | -1.61172941 |
| 101245736 | LOC101245736 | -1.354349573 | -1.404390255 | -1.955800196 |
| 101246083 | LOC101246083 | -1.902747893 | -1.950053608 | -1.974301154 |
| 101246479 | LOC101246479 | -2.483815777 | -2.024384159 | -7.483815777 |
| 101246621 | LOC101246621 | -1.709813988 | -1.966017901 | -2.430569355 |
| 101246713 | LOC101246713 | -3.089063169 | -4.607530258 | -4.647058622 |
| 101246823 | LOC101246823 | -1.245892365 | -1.678014233 | -1.3074862 |
| 101246978 | CYP96A48 | -1.157541277 | -1.372554168 | -1.249171752 |
| 101247174 | LOC101247174 | -1.439341259 | -1.450175065 | -2.039675774 |
| 101247499 | LOC101247499 | -1.419750653 | -2.73039294 | -3.997179481 |
| 101247612 | LOC101247612 | -1.247033927 | -1.516873511 | -1.506677744 |
| 101247834 | LOC101247834 | -1.245652328 | -1.178145508 | -1.7589919 |
| 101248058 | LOC101248058 | -1.491527027 | -1.212282394 | -2.172536215 |
| 101248093 | LOC101248093 | -1.098718051 | -1.20469042 | -1.086508993 |
| 101248236 | LOC101248236 | -5.339850003 | -2.695993813 | -5.339850003 |
| 101248427 | LOC101248427 | -2.303191532 | -1.935459748 | -1.765534746 |
| 101248557 | LOC101248557 | -4.380986654 | -3.493461383 | -4.24348313 |
| 101248705 | LOC101248705 | -1.115930112 | -1.475139223 | -1.182203331 |
| 101249335 | LOC101249335 | -1.222392421 | -1.017277991 | -1.567888987 |
| 101249482 | LOC101249482 | -2.169925001 | -1.146078259 | -1.491853096 |
| 101249503 | LOC101249503 | -1.671767328 | -2.083081741 | -2.290677161 |
| 101249517 | LOC101249517 | -1.470142099 | -1.567989422 | -1.90902634 |
| 101249827 | LOC101249827 | -1.306129385 | -1.153769233 | -1.444704615 |
| 101249836 | LOC101249836 | -1.983511877 | -1.5360529 | -2.488747185 |
| 101249884 | LOC101249884 | -2.050085231 | -1.518364752 | -1.631132682 |
| 101250126 | LOC101250126 | -1.627087976 | -1.364053571 | -2.031478231 |
| 101250416 | LOC101250416 | -2.887525271 | -2.039528364 | -2.887525271 |
| 101250466 | NAC2 | -1.605070074 | -1.20683493 | -1.718776648 |
| 101250517 | LOC101250517 | -2.84212034 | -1.951744831 | -2.723938914 |
| 101250637 | LOC101250637 | -1.60867857 | -1.851136614 | -1.51606742 |
| 101250825 | LOC101250825 | -1.109477586 | -1.241339976 | -2.080127406 |
| 101250945 | LOC101250945 | -1.127952731 | -2.611829107 | -1.569079459 |
| 101251007 | LOC101251007 | -1.154228815 | -1.249853418 | -1.077489371 |
| 101251053 | DXS2 | -1.310607559 | -1.180661386 | -1.479289393 |
| 101251577 | LOC101251577 | -2.386915696 | -2.469592719 | -3.298379021 |
| 101251636 | LOC101251636 | -1.434095835 | -2.596367264 | -2.288244969 |
| 101251651 | LOC101251651 | -1.115477217 | -1.512812715 | -2.326044203 |
| 101251725 | LOC101251725 | -2.057844075 | -1.539080064 | -1.803579878 |
| 101252298 | LOC101252298 | -1.112783404 | -2.66518723 | -1.77489115 |
| 101252313 | LOC101252313 | -1.772320871 | -3.220781371 | -2.290044034 |
| 101252437 | LOC101252437 | -1.404841732 | -2.510194732 | -2.647698256 |
| 101252458 | LOC101252458 | -1.868461009 | -2.412781525 | -2.308444866 |
| 101252493 | LOC101252493 | -1.638600464 | -2.425606741 | -3.215389034 |
| 101252520 | LOC101252520 | -8.252665432 | -5.252665432 | -8.252665432 |
| 101252561 | LOC101252561 | -1.313049944 | -1.880418384 | -1.432959407 |
| 101252569 | LOC101252569 | -2.816909229 | -2.26589406 | -2.288977673 |
| 101252722 | LOC101252722 | -1.405514725 | -2.028951374 | -2.04858018 |
| 101252788 | LOC101252788 | -1.326161816 | -1.011616684 | -1.441081254 |
| 101252820 | LOC101252820 | -3.339850003 | -2.803797103 | -2.003566615 |
| 101252847 | LOC101252847 | -1.003873009 | -1.413748803 | -1.928321976 |
| 101252867 | LOC101252867 | -1.721572263 | -1.29558422 | -2.893308883 |
| 101252930 | LOC101252930 | -2.763653613 | -2.142621857 | -2.028515371 |
| 101253176 | LOC101253176 | -2.089218898 | -3.133486877 | -4.506680416 |
| 101253384 | LOC101253384 | -1.75234082 | -2.621096287 | -4.237767647 |
| 101253390 | LOC101253390 | -1.834089333 | -2.663802527 | -3.222240198 |
| 101253456 | LOC101253456 | -2.294183104 | -2.162938571 | -2.986060809 |
| 101253548 | LOC101253548 | -1.147186077 | -1.49608122 | -1.39111166 |
| 101253673 | LOC101253673 | -1.050211446 | -1.086737322 | -1.912707922 |
| 101253721 | LOC101253721 | -3.459431619 | -2.408805546 | -1.743224585 |
| 101254060 | LOC101254060 | -1.796466606 | -1.584962501 | -1.702001922 |
| 101254394 | LOC101254394 | -1.315404971 | -1.488044357 | -2.770774565 |
| 101254501 | LOC101254501 | -1.766996589 | -1.81638559 | -2.965442778 |
| 101254625 | OFP2 | -1.665580961 | -3.61667136 | -3.716207034 |
| 101254641 | LOC101254641 | -1.748848586 | -1.361187525 | -1.873393684 |
| 101254739 | LOC101254739 | -1.014006858 | -1.952019971 | -1.064130337 |
| 101254867 | LOC101254867 | -5.129283017 | -2.070389328 | -3.714245518 |
| 101255257 | LOC101255257 | -1.103958633 | -1.73852322 | -2.356134914 |
| 101255270 | LOC101255270 | -1.036133786 | -1.48644676 | -1.089375807 |
| 101255514 | LOC101255514 | -2.658592075 | -1.65455091 | -1.912348667 |
| 101255537 | FKBP65 | -2.299560282 | -2 | -2.540568381 |
| 101255592 | LOC101255592 | -1.4639471 | -2.152003093 | -1.352301744 |
| 101255616 | LOC101255616 | -2.928107082 | -2.776103988 | -2.513069582 |
| 101255712 | LOC101255712 | -2.702302985 | -1.987425307 | -2.712879976 |
| 101255730 | LOC101255730 | -2.043721377 | -1.566243303 | -3.284729477 |
| 101255846 | LOC101255846 | -1.09646284 | -1.720314354 | -2.127489735 |
| 101256058 | LOC101256058 | -1.814968106 | -1.984893108 | -1.662965013 |
| 101256135 | LOC101256135 | -2.050626073 | -2.5360529 | -2.050626073 |
| 101256242 | LOC101256242 | -2.051625689 | -1.523343382 | -1.960329855 |
| 101256387 | LOC101256387 | -2.779609932 | -3.879145605 | -6.686500527 |
| 101256944 | LOC101256944 | -1.471006311 | -1.334214468 | -2.997179481 |
| 101257532 | CYP77B11 | -3.024166311 | -1.858580245 | -1.167530486 |
| 101257972 | LOC101257972 | -1.50389089 | -1.140007479 | -2 |
| 101258568 | LOC101258568 | -2.95419631 | -3.36923381 | -3.95419631 |
| 101258619 | LOC101258619 | -2.049753035 | -3.359081093 | -3.560714954 |
| 101258696 | JRE4 | -1.092549243 | -3.031148699 | -2.113610859 |
| 101258850 | LOC101258850 | -1.001007117 | -1.614458175 | -2.105444527 |
| 101258951 | LOC101258951 | -1.640918907 | -1.08061846 | -1.592824618 |
| 101259369 | LOC101259369 | -2.530514717 | -2.478047297 | -2.285402219 |
| 101259539 | LOC101259539 | -1.011012993 | -5.515357033 | -3.99452487 |
| 101259743 | LOC101259743 | -1.277663708 | -2.770165644 | -1.817666068 |
| 101259790 | LOC101259790 | -3.268488836 | -1.727920455 | -2.204358499 |
| 101260139 | LOC101260139 | -1.606612143 | -1.232086192 | -1.396665296 |
| 101260379 | LOC101260379 | -1.823474587 | -1.287980763 | -3.211745177 |
| 101260474 | LOC101260474 | -2.020464103 | -1.40155427 | -1.950074775 |
| 101260511 | LOC101260511 | -1.204091605 | -2.667063581 | -2.176077228 |
| 101260795 | LOC101260795 | -2.965234582 | -4.187627003 | -4.550197083 |
| 101261198 | LOC101261198 | -2.885334006 | -2.63691458 | -2.212725383 |
| 101261625 | LOC101261625 | -1.239187664 | -6.169925001 | -3.362570079 |
| 101262090 | LOC101262090 | -1.731183242 | -1.731183242 | -1.620151929 |
| 101262111 | LOC101262111 | -1.975196609 | -1.911066272 | -1.880039376 |
| 101262198 | LOC101262198 | -2.691561567 | -1.87036472 | -3.051605034 |
| 101262396 | LOC101262396 | -1.26960706 | -1.7589919 | -3.016488123 |
| 101262531 | LOC101262531 | -1.833783047 | -1.767440553 | -2.288977673 |
| 101263191 | LOC101263191 | -5.112700133 | -2.264703226 | -3.305345211 |
| 101263653 | LOC101263653 | -1.076783233 | -1.733861911 | -2.283099681 |
| 101264365 | LOC101264365 | -4.627786401 | -2.984818296 | -4.8172642 |
| 101264861 | LOC101264861 | -1.094327383 | -2.0061002 | -1.133855747 |
| 101264882 | LOC101264882 | -1.106041637 | -1.818759685 | -1.901221846 |
| 101264962 | LOC101264962 | -1.307667402 | -1.272620455 | -1.343586976 |
| 101265405 | LOC101265405 | -2.432959407 | -3.491853096 | -2.432959407 |
| 101265964 | LOC101265964 | -1.688055994 | -3.442943496 | -2.5360529 |
| 101266043 | LOC101266043 | -2.183345517 | -2.816288047 | -3.639410285 |
| 101266274 | LOC101266274 | -1.236700258 | -2.377562794 | -1.944146765 |
| 101266455 | LOC101266455 | -1.049803556 | -1.120043853 | -1.546602027 |
| 101266767 | LOC101266767 | -1.011012993 | -1.839246644 | -1.225137798 |
| 101267047 | LOC101267047 | -1.292781749 | -2.257157839 | -1.454838507 |
| 101267165 | LOC101267165 | -1.837728571 | -1.461580085 | -1.509674373 |
| 101267191 | LOC101267191 | -4.807354922 | -4.155278225 | -3.614709844 |
| 101267403 | LOC101267403 | -1.451379346 | -1.233199176 | -1.451379346 |
| 101267454 | LOC101267454 | -1.170810362 | -1.699112695 | -2.541373232 |
| 101267854 | LOC101267854 | -1.233097122 | -1.159780444 | -1.212729324 |
| 101267981 | LOC101267981 | -1.777607579 | -1.812372997 | -1.415037499 |
| 101268370 | LOC101268370 | -1.763853283 | -1.109181811 | -2.100136671 |
| 104644967 | LOC104644967 | -5.95419631 | -4.95419631 | -4.95419631 |
| 104645827 | LOC104645827 | -1.247927513 | -1.490497817 | -1.595850817 |
| 104646604 | LOC104646604 | -1.407982742 | -1.387023123 | -2.878009476 |
| 104648378 | LOC104648378 | -3.069708972 | -1.518467089 | -1.456457427 |
| 104648816 | LOC104648816 | -1.557995453 | -1.37540404 | -1.935362534 |
| 104648988 | LOC104648988 | -3.523561956 | -7.982993575 | -6.398031074 |
| 109120969 | LOC109120969 | -6.807354922 | -6.807354922 | -6.807354922 |
| 109121165 | LOC109121165 | -2.734340125 | -2.306246473 | -2.992137882 |
| 112940619 | LOC112940619 | -2.611214796 | -1.005098869 | -1.752697694 |
| 112940735 | LOC112940735 | -1.377157616 | -1.50589093 | -2.325318684 |
| 543540 | IAA3 | -1.145605322 | -2.093365906 | -2.358453971 |
| 543582 | EXP2 | -1.077194506 | -3.388609374 | -3.716899971 |
| 543615 | SIG1 | -2.454795209 | -1.981543095 | -1.909989834 |
| 543702 | HMGR | -1.662649911 | -1.240148892 | -1.777877721 |
| 543722 | CYCD3C2 | -1.996699392 | -1.904744342 | -1.986187906 |
| 543723 | CYCD3C3 | -1.674994702 | -2.476214837 | -2.9152578 |
| 543732 | LOC543732 | -1.436386819 | -1.127755547 | -1.882643049 |
| 543958 | SAR2 | -1.11418809 | -3.169683202 | -2.578207395 |
| 543959 | TPX2 | -3.169925001 | -4.491853096 | -3.321928095 |
| 544067 | TPRP-F1 | -3.027604461 | -1.378511623 | -2.505183427 |

**Table S3. Genes related with cell wall up-regulated by JA, SA and COR treatment**

| Treatment | Gene Symbol | Seq Description | log2 (T / MOCK) | Pvalue |
| --- | --- | --- | --- | --- |
| JA | LOC101257623 | galactinol--sucrose galactosyltransferase | 3.59 | 1.38E-21 |
|  | LOC101261450 | galactinol synthase 1 | 3.09 | 3.66E-04 |
|  | Wiv-1 | acid invertase | 2.71 | 2.46E-18 |
|  | TPS1 | trehalose-6-phosphate synthase | 1.85 | 3.09E-93 |
|  | LOC101260057 | beta-glucosidase BoGH3B | 1.28 | 1.01E-40 |
|  | LOC101266745 | bifunctional UDP-glucose 4-epimerase and UDP-xylose 4-epimerase 1 | 1.09 | 1.05E-11 |
| SA | LOC101257623 | galactinol--sucrose galactosyltransferase | 4.45 | 6.73E-45 |
|  | Wiv-1 | acid invertase | 3.57 | 1.34E-41 |
|  | TPS1 | trehalose-6-phosphate synthase | 2.53 | 1.45E-192 |
|  | LOC101259643 | beta-galactosidase 17 | 2.17 | 1.49E-208 |
|  | LOC101249633 | probable trehalose-phosphate phosphatase J | 2.16 | 2.11E-04 |
|  | LOC101257269 | alpha-galactosidase 3 | 2.07 | 7.31E-118 |
|  | LOC101256649 | hexokinase-3-like | 1.91 | 1.03E-32 |
|  | LOC101246803 | trehalose-phosphate phosphatase A | 1.70 | 2.70E-56 |
|  | CEL3 | endo-1,4-beta-glucanase | 1.48 | 0 |
|  | LOC101256154 | probable sucrose-phosphate synthase 4 | 1.42 | 6.71E-53 |
|  | SPS | sucrose-phosphate synthase | 1.39 | 7.58E-233 |
|  | LOC101257661 | alpha-amylase 3, chloroplastic | 1.34 | 1.36E-291 |
|  | LOC101260057 | beta-glucosidase BoGH3B | 1.30 | 1.08E-42 |
|  | LOC101253771 | ATP-dependent 6-phosphofructokinase 2 | 1.19 | 3.70E-15 |
|  | LOC101251982 | glucan endo-1,3-beta-glucosidase 6-like | 1.11 | 2.11E-04 |
|  | LOC101245938 | ATP-dependent 6-phosphofructokinase 5, chloroplastic-like | 1.08 | 3.65E-14 |
|  | LOC101266745 | bifunctional UDP-glucose 4-epimerase and UDP-xylose 4-epimerase 1 | 0.30 | 1.78E-82 |
| COR | LOC101246223 | beta-glucosidase 44-like | 7.62 | 2.35E-22 |
|  | LOC101258345 | probable xyloglucan endotransglucosylase/hydrolase protein 23 | 5.91 | 2.49E-04 |
|  | LOC101267720 | sucrose synthase-like | 4.32 | 1.87E-27 |
|  | AROGP2 | polygalacturonase non-catalytic subunit AroGP2 | 3.52 | 7.24E-08 |
|  | XET2 | xyloglucan endotransglycosylase LeXET2 | 2.75 | 0 |
|  | LOC101249633 | probable trehalose-phosphate phosphatase J | 2.37 | 2.53E-05 |
|  | Frk1 | fructokinase | 2.12 | 3.46E-09 |
|  | XET-B1 | xyloglucan endo-transglycosylase | 1.86 | 1.32E-24 |
|  | Wiv-1 | acid invertase | 1.84 | 3.83E-07 |
|  | LOC101246803 | trehalose-phosphate phosphatase A | 1.68 | 2.60E-54 |
|  | PR-P2 | pathogenesis-related protein P2 | 1.63 | 1.50E-06 |
|  | XTH7 | xyloglucan endotransglucosylase-hydrolase XTH7 | 1.47 | 6.81E-237 |
|  | LOC101055611 | sucrose-phosphate synthase A2 | 1.45 | 2.48E-08 |
|  | LOC101256341 | aldose 1-epimerase-like | 1.39 | 5.33E-05 |
|  | AgpL1 | ADP-glucose pyrophosphorylase large subunit | 1.36 | 1.69E-36 |
|  | LOC101245938 | ATP-dependent 6-phosphofructokinase 5, chloroplastic-like | 1.32 | 6.87E-22 |
|  | LOC104645805 | beta-glucosidase 18 | 1.29 | 4.32E-10 |
|  | LOC543961 | sucrose synthase | 1.24 | 1.24E-15 |
|  | LOC543511 | probable xyloglucan endotransglucosylase/hydrolase protein 27 | 1.24 | 1.78E-82 |
|  | LOC101264994 | alpha-glucosidase-like | 1.06 | 1.47E-07 |
|  | LOC101244979 | probable trehalase | 1.06 | 7.04E-07 |
|  | LOC101244370 | endoglucanase 25-like | 1.05 | 3.64E-88 |
|  | XTH1 | endo-xyloglucan transferase | 1.04 | 9.96E-113 |
|  | LIN8 | invertase 8 | 1.03 | 8.79E-24 |
|  | AGP-S2 | ADP-glucose pyrophosphorylase large subunit | 1.01 | 1.10E-46 |
|  | LOC101250326 | alpha,alpha-trehalose-phosphate synthase [UDP-forming] 5 | 1.01 | 4.17E-23 |
|  | LOC101260787 | pectinesterase-like | 1.01 | 2.61E-185 |
|  | TBG1 | beta-galactosidase | 1.00 | 1.12E-20 |
|  | TPS1 | trehalose-6-phosphate synthase | 0.32 | 0 |

**Table S4. Genes related with cell wall down-regulated by JA, SA and COR treatment**

| Treatment | Gene Symbol | Seq Description | log2 (T / MOCK) | Pvalue |
| --- | --- | --- | --- | --- |
| JA | LOC101259175 | beta-amylase 3, chloroplastic-like | -1.01 | 2.09E-06 |
|  | LOC101256510 | beta-glucosidase 40 | -1.04 | 1.39E-11 |
|  | LOC101259862 | glucan endo-1,3-beta-glucosidase 2-like | -1.04 | 3.57E-08 |
|  | LOC100191127 | beta-glucosidase 01 | -1.08 | 2.59E-86 |
|  | LOC101257723 | endoglucanase 2-like | -1.11 | 1.46E-17 |
|  | LOC101249596 | probable starch synthase 4, chloroplastic/amyloplastic | -1.15 | 5.70E-17 |
|  | LOC101257526 | beta-glucosidase 18 | -1.16 | 1.64E-26 |
|  | LOC101250269 | glucan endo-1,3-beta-glucosidase 1 | -1.20 | 1.61E-19 |
|  | LOC101246556 | glucan endo-1,3-beta-glucosidase 6 | -1.22 | 1.05E-11 |
|  | LOC101255377 | sucrose synthase 5-like | -1.32 | 1.22E-37 |
|  | LOC101268012 | 1,4-alpha-glucan-branching enzyme 3, chloroplastic/amyloplastic | -1.39 | 6.66E-13 |
|  | LOC101267699 | glucan endo-1,3-beta-glucosidase 3 | -1.54 | 3.61E-23 |
|  | LOC101249741 | isoamylase 1, chloroplastic | -1.55 | 2.74E-49 |
|  | CEL8 | endo-beta-1,4-D-glucanase | -1.65 | 2.09E-157 |
|  | LOC101055573 | sucrose-phosphate synthase B | -1.75 | 9.61E-07 |
|  | CEL7 | endo-1,4-beta-D-glucanase | -2.10 | 7.13E-18 |
|  | LOC101264195 | beta-fructofuranosidase, insoluble isoenzyme CWINV1 | -2.63 | 6.76E-05 |
|  | LOC101247302 | endoglucanase 1 | -3.32 | 1.33E-58 |
|  | LOC101249034 | probable hexokinase-like 2 protein | -3.66 | 1.48E-07 |
| SA | LOC101266782 | sucrose synthase 7-like | -1.11 | 1.17E-26 |
|  | LOC101255377 | sucrose synthase 5-like | -1.13 | 3.60E-30 |
|  | LOC543698 | beta-mannosidase | -1.30 | 3.44E-291 |
|  | Cellulase | endo-1,4-beta-glucanase precursor | -1.33 | 2.05E-37 |
|  | LOC101268012 | 1,4-alpha-glucan-branching enzyme 3, chloroplastic/amyloplastic | -1.53 | 9.18E-15 |
|  | LOC101257526 | beta-glucosidase 18 | -1.63 | 7.79E-43 |
|  | CEL8 | endo-beta-1,4-D-glucanase | -1.76 | 1.02E-174 |
|  | LOC101266643 | beta-glucosidase BoGH3B-like | -2.00 | 1.67E-04 |
|  | CEL7 | endo-1,4-beta-D-glucanase | -2.35 | 1.31E-20 |
|  | LOC101249034 | probable hexokinase-like 2 protein | -2.93 | 2.19E-06 |
|  | LOC101247302 | endoglucanase 1 | -3.34 | 9.80E-60 |
| COR | LOC101266745 | bifunctional UDP-glucose 4-epimerase and UDP-xylose 4-epimerase 1 | -0.17 | 1.78E-82 |
|  | EXP2 | expansin | -1.08 | 1.06E-95 |
|  | CEL2 | endo-1,4-beta-glucanase precursor | -1.20 | 5.83E-113 |
|  | XET4 | xyloglucan endotransglycosylase | -1.47 | 3.57E-05 |
|  | LOC101247960 | pectinesterase 2 | -1.66 | 3.75E-13 |
|  | LOC101253894 | probable xyloglucan endotransglucosylase/hydrolase protein 33 | -2.16 | 1.89E-04 |
|  | LOC101263843 | stachyose synthase | -2.81 | 1.52E-04 |

**Table S5. Genes involved in plant hormone signal transduction pathway up-regulated by JA, SA and COR**

| Treatment | Gene Symbol | Seq Description | log2 (T / MOCK) | Pvalue |
| --- | --- | --- | --- | --- |
| JA | LOC101263193 | protein TIFY 10b-like | 2.88 | 9.35E-258 |
|  | LOC100191111 | PR1 protein | 2.29 | 4.03E-11 |
|  | Prg1 | Pto-responsive gene 1 protein | 2.15 | 0 |
|  | LOC101258963 | abscisic acid receptor PYL4 | 2.06 | 9.02E-29 |
|  | LOC101247936 | protein TIFY 10b-like | 1.81 | 1.76E-169 |
|  | LOC101249794 | protein phosphatase 2C 51-like | 1.47 | 2.75E-53 |
|  | NML2 | NIM1-like protein 2 | 1.41 | 1.01E-72 |
|  | JAR1 | jasmonic acid-amido synthetase JAR1 | 1.32 | 7.68E-127 |
|  | LOC101250433 | serine/threonine-protein kinase SAPK3 | 1.30 | 2.25E-48 |
|  | JAZ2 | jasmonate ZIM-domain protein 2 | 1.29 | 4.05E-14 |
|  | ETR4 | ethylene receptor ETR4 | 1.15 | 6.04E-06 |
|  | LOC109119038 | BRI1 kinase inhibitor 1 | 1.14 | 7.33E-57 |
|  | LOC101262506 | histidine kinase 4 | 1.14 | 1.22E-07 |
|  | LOC101263766 | ABSCISIC ACID-INSENSITIVE 5-like protein 7 | 1.14 | 2.51E-33 |
|  | LOC101253938 | histidine-containing phosphotransfer protein 1 | 1.08 | 5.77E-07 |
|  | LOC101255313 | auxin-responsive protein SAUR71 | 1.05 | 5.31E-06 |
|  | LOC101262109 | BRI1 kinase inhibitor 1 | 0.53 | 1.16E-17 |
| SA | LOC101257321 | auxin-responsive protein SAUR71-like | 7.04 | 1.03E-06 |
|  | LOC101258345 | probable xyloglucan endotransglucosylase/hydrolase protein 23 | 5.95 | 1.28E-04 |
|  | LOC100191111 | PR1 protein | 4.49 | 6.98E-86 |
|  | GH3-4 | putative indole-3-acetic acid amido synthetase | 2.80 | 3.96E-12 |
|  | PR1b1 | pathogenesis-related leaf protein 6 | 2.71 | 1.64E-26 |
|  | LOC109119038 | BRI1 kinase inhibitor 1 | 2.53 | 0 |
|  | LOC101262109 | BRI1 kinase inhibitor 1 | 2.34 | 2.86E-55 |
|  | LOC101249794 | protein phosphatase 2C 51-like | 2.33 | 7.81E-179 |
|  | JAZ2 | jasmonate ZIM-domain protein 2 | 2.13 | 1.34E-47 |
|  | LOC101266334 | two-component response regulator ARR1 | 2.01 | 2.16E-137 |
|  | NML2 | NIM1-like protein 2 | 1.85 | 7.85E-152 |
|  | SRG1 | salt responsive protein 1 | 1.74 | 7.19E-200 |
|  | LOC101263193 | protein TIFY 10b-like | 1.73 | 4.53E-64 |
|  | GH3-5 | putative indole-3-acetic acid-amido synthetase GH3.5 | 1.64 | 2.86E-89 |
|  | LOC101255900 | histidine kinase 2 | 1.62 | 1.91E-175 |
|  | LOC104645438 | auxin-responsive protein SAUR22-like | 1.57 | 2.52E-06 |
|  | ABI2 | protein phosphatase 2C ABI2 | 1.54 | 2.72E-127 |
|  | LOC101252303 | transcription factor PIF4 | 1.48 | 0 |
|  | EIL2 | protein EIN3-like2 | 1.48 | 2.12E-215 |
|  | LOC101265384 | DELLA protein GAI | 1.48 | 2.60E-04 |
|  | SRK2C | SNF1-related kinase | 1.47 | 4.80E-44 |
|  | LOC101055555 | IAA35 | 1.41 | 5.83E-27 |
|  | SERK3A | somatic embryogenesis receptor kinase 3A | 1.30 | 2.11E-56 |
|  | LOC101250433 | serine/threonine-protein kinase SAPK3 | 1.25 | 4.75E-45 |
|  | LOC101263766 | ABSCISIC ACID-INSENSITIVE 5-like protein 7 | 1.22 | 1.43E-39 |
|  | TGA5 | transcription factor TGA5 | 1.21 | 5.79E-47 |
|  | SERK3B | somatic embryogenesis receptor kinase 3B | 1.14 | 2.73E-35 |
|  | EIL4 | protein ETHYLENE-INSENSITIVE 3-like 4 | 1.12 | 2.05E-92 |
|  | LOC101247936 | protein TIFY 10b-like | 1.11 | 9.89E-52 |
|  | LOC101055583 | small auxin-up protein 58 | 1.10 | 1.47E-13 |
|  | LOC101267105 | ethylene-responsive transcription factor 1B | 1.09 | 3.52E-06 |
|  | JAR1 | jasmonic acid-amido synthetase JAR1 | 1.07 | 1.92E-80 |
|  | ETR4 | ethylene receptor ETR4 | 1.05 | 5.07E-05 |
|  | LAX4 | auxin transporter-like protein 4 | 1.05 | 8.53E-10 |
|  | LOC543567 | serine/threonine-protein kinase BSK7 | 1.04 | 1.06E-67 |
|  | LOC101260897 | BES1/BZR1 homolog protein 2 | 1.04 | 1.78E-111 |
|  | LOC101251432 | serine/threonine-protein kinase SRK2I | 1.02 | 9.93E-212 |
|  | LOC101258963 | abscisic acid receptor PYL4 | 1.01 | 4.11E-06 |
|  | LOC606712 | ethylene-responsive transcription factor 1 | 1.00 | 1.51E-05 |
| COR | LOC101258345 | probable xyloglucan endotransglucosylase/hydrolase protein 23 | 5.91 | 2.49E-04 |
|  | LOC101248225 | histidine-containing phosphotransfer protein 4-like | 3.60 | 5.83E-06 |
|  | BOP1 | BLADE-ON-PETIOLE protein BOP1 | 2.08 | 9.49E-06 |
|  | LOC101250944 | abscisic acid receptor PYL4 | 1.78 | 3.08E-12 |
|  | LOC101253982 | transcription factor TGA1 | 1.65 | 9.11E-62 |
|  | BOP2 | BLADE-ON-PETIOLE protein BOP2 | 1.62 | 2.28E-08 |
|  | LOC100191111 | PR1 protein | 1.46 | 2.76E-04 |
|  | SRK2C | SNF1-related kinase | 1.36 | 4.85E-37 |
|  | LOC101249950 | ETHYLENE INSENSITIVE 3-like 3 protein | 1.23 | 1.23E-05 |
|  | LOC101262506 | histidine kinase 4 | 1.09 | 3.97E-07 |
|  | LOC101255313 | auxin-responsive protein SAUR71 | 1.07 | 2.39E-06 |
|  | LOC101267127 | abscisic acid receptor PYR1 | 1.07 | 6.06E-22 |
|  | JAZ2 | jasmonate ZIM-domain protein 2 | 1.05 | 2.18E-09 |
|  | LOC101055583 | small auxin-up protein 58 | 1.03 | 5.81E-12 |

**Table S6. Genes involved in Plant hormone signal transduction pathway down-regulated by JA, SA and COR**

| Treatment | Gene Symbol | Seq Description | log2 (T / MOCK) | Pvalue |
| --- | --- | --- | --- | --- |
| JA | PR1b1 | pathogenesis-related leaf protein 6 | -0.29 | 3.75E-16 |
|  | GH3-1 | jasmonic acid-amido synthetase JAR1-like | -1.03 | 6.94E-55 |
|  | LOC101258071 | protein phosphatase 2C | -1.03 | 3.44E-20 |
|  | LOC101267355 | transcription factor PIF3 | -1.03 | 2.08E-07 |
|  | GID1ac | gibberellin receptor GID1ac | -1.08 | 6.07E-08 |
|  | LOC104645434 | auxin-induced protein 15A-like | -1.10 | 2.74E-08 |
|  | IAA2 | auxin-responsive protein IAA2 | -1.15 | 1.16E-04 |
|  | LOC101261086 | serine/threonine-protein kinase SAPK2 | -1.15 | 1.07E-07 |
|  | BOP3 | BLADE-ON-PETIOLE protein BOP3 | -1.16 | 1.51E-06 |
|  | LOC101248778 | protein phosphatase 2C 37 | -1.17 | 0 |
|  | LOC101253234 | auxin-responsive protein SAUR50-like | -1.21 | 3.79E-04 |
|  | LOC101248058 | two-component response regulator ORR9 | -1.21 | 2.17E-24 |
|  | LOC109118704 | auxin-induced protein 15A-like | -1.34 | 2.45E-15 |
|  | ARF9 | auxin response factor 9 | -1.35 | 5.44E-05 |
|  | PP2C-2 | protein phosphatase 2C AHG3 homolog | -1.43 | 2.56E-47 |
|  | IAA19 | auxin-responsive protein IAA19 | -1.44 | 1.61E-05 |
|  | LOC101247174 | two-component response regulator ARR9 | -1.45 | 1.15E-41 |
|  | LOC101250316 | auxin-responsive protein SAUR50 | -1.52 | 1.28E-08 |
|  | LOC101265701 | two-component response regulator ORR3-like | -1.53 | 1.19E-42 |
|  | LOC101251725 | auxin-responsive protein SAUR50 | -1.54 | 7.95E-10 |
|  | LOC101267105 | ethylene-responsive transcription factor 1B | -1.54 | 8.18E-05 |
|  | ARF5 | auxin response factor 5 | -1.76 | 3.75E-16 |
|  | LOC101246270 | auxin-responsive protein SAUR36-like | -1.78 | 6.67E-08 |
|  | LAX2 | auxin transporter-like protein 2 | -1.81 | 3.82E-22 |
|  | GH3-8 | protein GRETCHEN HAGEN 3-8 | -1.88 | 7.06E-169 |
|  | CYCD3c2 | D-type cyclin-2 | -1.90 | 1.60E-66 |
|  | LOC101249503 | auxin-responsive protein SAUR50-like | -2.08 | 1.16E-17 |
|  | IAA3 | IAA3 protein | -2.09 | 2.17E-38 |
|  | LOC101252930 | auxin-responsive protein SAUR50-like | -2.14 | 3.50E-20 |
|  | LOC101249950 | ETHYLENE INSENSITIVE 3-like 3 protein | -2.32 | 9.85E-06 |
|  | CYCD3c3 | D-type cyclin-3 | -2.48 | 2.03E-47 |
|  | LOC101253384 | two-component response regulator ARR17 | -2.62 | 1.14E-06 |
|  | LOC101055550 | IAA22 | -2.67 | 1.76E-23 |
|  | LOC101247499 | auxin-responsive protein SAUR50 | -2.73 | 1.46E-13 |
|  | LOC104645436 | auxin-responsive protein SAUR21-like | -3.21 | 5.30E-09 |
|  | IAA21 | AUX/IAA 2 | -3.78 | 5.82E-10 |
|  | GH3-15 | indole-3-acetic acid-amido synthetase GH3.6-like | -5.39 | 8.46E-06 |
| SA | ARF5 | auxin response factor 5 | -1.09 | 1.73E-08 |
|  | LOC101263158 | auxin-responsive protein SAUR32 | -1.13 | 4.48E-36 |
|  | IAA2 | auxin-responsive protein IAA2 | -1.20 | 3.73E-04 |
|  | IAA19 | auxin-responsive protein IAA19 | -1.59 | 2.80E-06 |
|  | LOC101260027 | cyclin-D3-3 | -1.70 | 2.34E-28 |
|  | LOC101251725 | auxin-responsive protein SAUR50 | -1.80 | 4.04E-12 |
|  | LOC101250316 | auxin-responsive protein SAUR50 | -1.86 | 3.39E-11 |
|  | LOC101246270 | auxin-responsive protein SAUR36-like | -1.95 | 1.72E-08 |
|  | CYCD3c2 | D-type cyclin-2 | -1.99 | 1.97E-70 |
|  | LOC101252930 | auxin-responsive protein SAUR50-like | -2.03 | 5.25E-19 |
|  | LOC101247174 | two-component response regulator ARR9 | -2.04 | 7.65E-67 |
|  | LOC101248058 | two-component response regulator ORR9 | -2.17 | 1.58E-54 |
|  | LOC101258256 | auxin-responsive protein SAUR50 | -2.20 | 1.80E-04 |
|  | LOC104645436 | auxin-responsive protein SAUR21-like | -2.21 | 1.81E-06 |
|  | LOC101265701 | two-component response regulator ORR3-like | -2.22 | 1.59E-70 |
|  | LOC101249503 | auxin-responsive protein SAUR50-like | -2.29 | 6.57E-20 |
|  | IAA3 | IAA3 protein | -2.36 | 1.96E-44 |
|  | LOC101260711 | two-component response regulator ARR15 | -2.54 | 2.90E-16 |
|  | CYCD3c3 | D-type cyclin-3 | -2.92 | 4.09E-57 |
|  | LOC101055550 | IAA22 | -3.05 | 4.69E-27 |
|  | IAA21 | AUX/IAA 2 | -3.41 | 2.42E-09 |
|  | LOC101247499 | auxin-responsive protein SAUR50 | -4.00 | 7.30E-19 |
|  | LOC101253384 | two-component response regulator ARR17 | -4.24 | 1.31E-09 |
| COR | LOC101262109 | BRI1 kinase inhibitor 1 | -0.12 | 1.16E-17 |
|  | LOC101249794 | protein phosphatase 2C 51-like | -1.01 | 4.91E-12 |
|  | GH3-8 | protein GRETCHEN HAGEN 3-8 | -1.01 | 4.30E-67 |
|  | LOC104645434 | auxin-induced protein 15A-like | -1.11 | 1.47E-08 |
|  | LOC109118704 | auxin-induced protein 15A-like | -1.11 | 6.95E-12 |
|  | IAA3 | IAA3 protein | -1.15 | 7.60E-17 |
|  | LOC101252689 | two-component response regulator ORR26 | -1.17 | 5.82E-215 |
|  | IAA2 | auxin-responsive protein IAA2 | -1.22 | 2.48E-04 |
|  | IAA15 | auxin-regulated IAA15 | -1.23 | 1.01E-22 |
|  | LOC101247499 | auxin-responsive protein SAUR50 | -1.42 | 1.28E-06 |
|  | LOC101247174 | two-component response regulator ARR9 | -1.44 | 1.70E-41 |
|  | LOC101248058 | two-component response regulator ORR9 | -1.49 | 4.95E-33 |
|  | LOC101249503 | auxin-responsive protein SAUR50-like | -1.67 | 1.44E-13 |
|  | CYCD3c3 | D-type cyclin-3 | -1.67 | 1.96E-29 |
|  | LOC101253384 | two-component response regulator ARR17 | -1.75 | 1.37E-04 |
|  | IAA19 | auxin-responsive protein IAA19 | -1.81 | 3.10E-07 |
|  | LOC101055550 | IAA22 | -1.82 | 2.61E-15 |
|  | CYCD3c2 | D-type cyclin-2 | -2.00 | 2.33E-70 |
|  | LOC101251725 | auxin-responsive protein SAUR50 | -2.06 | 5.66E-14 |
|  | IAA21 | AUX/IAA 2 | -2.08 | 5.29E-06 |
|  | LOC101055556 | IAA36 | -2.76 | 1.87E-05 |
|  | LOC101252930 | auxin-responsive protein SAUR50-like | -2.76 | 2.88E-27 |
|  | LOC101253938 | histidine-containing phosphotransfer protein 1 | -2.81 | 2.20E-11 |
|  | LOC109119038 | BRI1 kinase inhibitor 1 | -5.90 | 3.10E-147 |

**Table S7. Genes involved in secondary metabolism up-regulated by three treatments**

| Treatment | Gene Symbol | Seq Description | log2 (T / MOCK) | Pvalue |
| --- | --- | --- | --- | --- |
| JA | LOC112940330 | lignin-forming anionic peroxidase | 9.56 | 3.05E-53 |
|  | LOC101253859 | agmatine hydroxycinnamoyltransferase 1-like | 4.41 | 2.93E-41 |
|  | AADC2 | aromatic amino acid decarboxylase 2 | 4.01 | 4.22E-30 |
|  | LOC101265187 | caffeoyl-CoA O-methyltransferase-like | 3.40 | 1.22E-174 |
|  | LOC101246783 | lignin-forming anionic peroxidase | 3.16 | 1.27E-25 |
|  | LOC101260610 | acetyl-CoA-benzylalcohol acetyltransferase | 3.08 | 0 |
|  | LOC101259064 | polyphenol oxidase F, chloroplastic | 3.06 | 0 |
|  | LOC101262081 | linoleate 13S-lipoxygenase 2-1, chloroplastic | 2.69 | 3.74E-11 |
|  | LOC112940337 | lignin-forming anionic peroxidase-like | 2.60 | 5.94E-15 |
|  | LOC101258774 | polyphenol oxidase B, chloroplastic-like | 2.43 | 1.99E-31 |
|  | 4CL | 4-coumarate--CoA ligase | 2.28 | 0 |
|  | LOC101256271 | shikimate O-hydroxycinnamoyltransferase | 2.25 | 2.88E-207 |
|  | LOC101265690 | caffeoyl-CoA O-methyltransferase | 2.10 | 1.71E-04 |
|  | LOC101258529 | peroxidase P7-like | 2.10 | 6.39E-14 |
|  | LOC101255659 | probable amidase At4g34880 | 1.92 | 1.55E-44 |
|  | LOC101243631 | phenylalanine ammonia-lyase-like | 1.87 | 6.50E-258 |
|  | LOC101244682 | peroxidase P7-like | 1.86 | 1.73E-05 |
|  | LOC101244196 | trans-cinnamate 4-monooxygenase | 1.76 | 7.30E-28 |
|  | LOC101261704 | fumarylacetoacetase | 1.60 | 1.99E-05 |
|  | Twi1 | scopoletin glucosyltransferase | 1.54 | 4.98E-239 |
|  | PAL3 | phenylalanine ammonia-lyase 3 | 1.53 | 0 |
|  | LOC101248210 | 4-coumarate--CoA ligase | 1.45 | 5.74E-83 |
|  | LOC101243656 | phenylalanine ammonia-lyase 6 | 1.44 | 0 |
|  | PAL5 | phenylalanine ammonia-lyase 5 | 1.37 | 1.20E-37 |
|  | LOC101246092 | p-coumaroyl quinate/shikimate 3-hydroxylase | 1.35 | 3.67E-61 |
|  | LOC101260057 | beta-glucosidase BoGH3B | 1.28 | 1.01E-40 |
|  | LOC101253684 | peroxidase 12 | 1.27 | 1.40E-18 |
|  | CEVI-16 | peroxidase | 1.22 | 1.81E-04 |
|  | CEVI-1 | peroxidase | 1.19 | 2.15E-04 |
|  | LOC101244246 | peroxidase 63 | 1.19 | 2.58E-45 |
|  | LOC101253556 | agmatine hydroxycinnamoyltransferase 1-like | 1.13 | 2.96E-04 |
|  | LOC101254287 | caffeoylshikimate esterase | 1.05 | 3.39E-82 |
|  | LOC101253032 | caffeoyl-CoA O-methyltransferase 5 | 1.03 | 2.26E-06 |
|  | LOC101244496 | trans-cinnamate 4-monooxygenase | 1.02 | 3.67E-13 |
|  | LoxC | lipoxygenase | 0.76 | 5.83E-07 |
| SA | LOC112940330 | lignin-forming anionic peroxidase | 9.26 | 1.49E-43 |
|  | LOC101265187 | caffeoyl-CoA O-methyltransferase-like | 4.11 | 0 |
|  | LOC112940337 | lignin-forming anionic peroxidase-like | 3.96 | 2.70E-52 |
|  | LOC101246783 | lignin-forming anionic peroxidase | 3.05 | 4.13E-23 |
|  | AADC2 | aromatic amino acid decarboxylase 2 | 3.03 | 6.98E-13 |
|  | LOC101260610 | acetyl-CoA-benzylalcohol acetyltransferase | 2.96 | 0 |
|  | LOC101243631 | phenylalanine ammonia-lyase-like | 2.66 | 0 |
|  | LOC101244246 | peroxidase 63 | 2.42 | 1.72E-281 |
|  | CEVI-1 | peroxidase | 2.22 | 1.49E-16 |
|  | LOC101253859 | agmatine hydroxycinnamoyltransferase 1-like | 2.13 | 9.95E-06 |
|  | Twi1 | scopoletin glucosyltransferase | 1.38 | 2.26E-181 |
|  | LOC101265690 | caffeoyl-CoA O-methyltransferase | 1.35 | 0.0361702 |
|  | LOC101260057 | beta-glucosidase BoGH3B | 1.30 | 1.08E-42 |
|  | PAL3 | phenylalanine ammonia-lyase 3 | 1.21 | 0 |
|  | LOC101255659 | probable amidase At4g34880 | 1.20 | 9.81E-15 |
|  | LOC101256271 | shikimate O-hydroxycinnamoyltransferase | 1.09 | 6.11E-34 |
|  | LOC101244682 | peroxidase P7-like | 1.06 | 0.0354158 |
|  | 4CL | 4-coumarate--CoA ligase | 1.01 | 2.61E-106 |
|  | PPO | polyphenol oxidase B, chloroplastic-like | 0.83 | 0.00254768 |
|  | CEVI-16 | peroxidase | 0.64 | 6.72E-05 |
|  | LOC101253556 | agmatine hydroxycinnamoyltransferase 1-like | 0.58 | 9.95E-06 |
|  | LOC101253032 | caffeoyl-CoA O-methyltransferase 5 | 0.56 | 2.88E-06 |
|  | LOC101244196 | trans-cinnamate 4-monooxygenase | 0.55 | 0.00479536 |
|  | LOC101244496 | trans-cinnamate 4-monooxygenase | 0.35 | 0.0270254 |
|  | LOC101253684 | peroxidase 12 | 0.33 | 0.0474626 |
|  | LOC101259064 | polyphenol oxidase F, chloroplastic | 0.31 | 8.99E-10 |
|  | LOC101248210 | 4-coumarate--CoA ligase | 0.29 | 0.00155368 |
|  | LOC101258529 | peroxidase P7-like | 0.24 | 0.52948 |
|  | LOC100191127 | beta-glucosidase 01 | 0.17 | 1.30E-04 |
| COR | LOC112940330 | lignin-forming anionic peroxidase | 6.57 | 2.46E-07 |
|  | CEVI-1 | peroxidase | 3.46 | 5.15E-59 |
|  | LOC101253859 | agmatine hydroxycinnamoyltransferase 1-like | 2.82 | 1.46E-10 |
|  | AADC2 | aromatic amino acid decarboxylase 2 | 2.63 | 9.62E-09 |
|  | LOC101265690 | caffeoyl-CoA O-methyltransferase | 2.46 | 2.95E-06 |
|  | LOC101259064 | polyphenol oxidase F, chloroplastic | 2.39 | 0 |
|  | LOC101253684 | peroxidase 12 | 2.03 | 5.96E-59 |
|  | LOC101243631 | phenylalanine ammonia-lyase-like | 1.93 | 1.32E-282 |
|  | LOC101255659 | probable amidase At4g34880 | 1.81 | 1.81E-38 |
|  | LOC101264847 | histidine decarboxylase-like | 1.75 | 1.21E-16 |
|  | LOC101244196 | trans-cinnamate 4-monooxygenase | 1.25 | 9.53E-13 |
|  | LOC101244682 | peroxidase P7-like | 1.12 | 5.42E-16 |
|  | LOC101244246 | peroxidase 63 | 1.10 | 4.27E-38 |
|  | LOC101260610 | acetyl-CoA-benzylalcohol acetyltransferase | 1.07 | 1.10E-33 |
|  | LOC101244496 | trans-cinnamate 4-monooxygenase | 0.94 | 5.94E-11 |
|  | LOC101246783 | lignin-forming anionic peroxidase | 0.85 | 0.0673656 |
|  | LOC101260057 | beta-glucosidase BoGH3B | 0.84 | 5.42E-16 |
|  | PPO | polyphenol oxidase B, chloroplastic-like | 0.79 | 0.00399436 |
|  | LOC101265187 | caffeoyl-CoA O-methyltransferase-like | 0.74 | 1.46E-04 |
|  | LOC101248210 | 4-coumarate--CoA ligase | 0.67 | 6.47E-15 |
|  | PAL3 | phenylalanine ammonia-lyase 3 | 0.64 | 5.54E-288 |
|  | LOC100191129 | anthocyanin acyltransferase | 0.53 | 0.00953992 |
|  | CEVI16 | peroxidase | 0.46 | 2.53E-04 |
|  | Twi1 | scopoletin glucosyltransferase | 0.36 | 3.47E-10 |
|  | LOC101243656 | phenylalanine ammonia-lyase 6 | 0.28 | 5.43E-11 |
|  | LOC101254287 | caffeoylshikimate esterase | 0.26 | 3.54E-05 |
|  | LOC101258529 | peroxidase P7-like | 0.25 | 5.42E-16 |
|  | 4CL | 4-coumarate--CoA ligase | 0.20 | 1.82E-04 |
|  | LOC101261193 | anthocyanidin 3-O-glucosyltransferase 5 | 0.18 | 0.514194 |
|  | LOC101256271 | shikimate O-hydroxycinnamoyltransferase | 0.16 | 0.1180854 |
|  | LOC101255909 | 4-coumarate--CoA ligase-like 6 | 0.12 | 0.524536 |
|  | LOC100191127 | beta-glucosidase 01 | 0.10 | 0.0290146 |
|  | LOC101246092 | p-coumaroyl quinate/shikimate 3-hydroxylase | 0.03 | 5.42E-16 |
|  | PAL5 | phenylalanine ammonia-lyase 5 | 0.02 | 1.20E-37 |

**Table S8. Genes involved in secondary metabolism down-regulated by three treatments**

| Treatment | Gene Symbol | Seq Description | log2 (T / MOCK) | Pvalue |
| --- | --- | --- | --- | --- |
| JA | CHI1 | chalcone--flavonone isomerase 1 | -0.72 | 3.14E-07 |
|  | LOC101256510 | beta-glucosidase 40 | -1.04 | 1.39E-11 |
|  | LOC101255909 | 4-coumarate--CoA ligase-like 6 | -1.04 | 9.77E-06 |
|  | LOC100191127 | beta-glucosidase 01 | -1.08 | 2.59E-86 |
|  | CHS2 | chalcone synthase | -1.12 | 2.37E-16 |
|  | LOC101257526 | beta-glucosidase 18 | -1.16 | 1.64E-26 |
|  | LOC101247834 | caffeic acid 3-O-methyltransferase-like | -1.18 | 1.50E-06 |
|  | F3H | flavanone 3-dioxygenase | -1.21 | 4.56E-09 |
|  | CHS1 | chalcone synthase | -1.24 | 0.01276622 |
|  | LOC101263431 | tryptophan decarboxylase TDC1-like | -1.27 | 5.69E-05 |
|  | LOC101265606 | berberine bridge enzyme-like 15 | -1.29 | 3.38E-05 |
|  | F3'5'H | flavonoid 35 hydroxylase | -1.32 | 6.35E-17 |
|  | LOC101265461 | histidine decarboxylase-like | -1.44 | 6.22E-05 |
|  | LOC101266674 | probable aminotransferase TAT2 | -1.64 | 1.63E-23 |
|  | LOC101261193 | anthocyanidin 3-O-glucosyltransferase 5 | -1.85 | 3.52E-06 |
|  | LOC100191129 | anthocyanin acyltransferase | -2.11 | 6.30E-10 |
|  | LOC101264847 | histidine decarboxylase-like | -2.25 | 7.29E-08 |
|  | TAP2 | Suberization-associated anionic peroxidase 2 | -2.56 | 2.88E-06 |
|  | LOC101248236 | peroxidase 44-like | -2.70 | 3.78E-07 |
|  | LOC101253862 | primary amine oxidase 1 | -3.29 | 2.44E-04 |
|  | TPX2 | peroxidase | -4.49 | 2.72E-07 |
| SA | LOC101254287 | caffeoylshikimate esterase | -0.12 | 6.72E-05 |
|  | LOC101262081 | linoleate 13S-lipoxygenase 2-1, chloroplastic | -0.19 | 2.88E-06 |
|  | LoxC | lipoxygenase | -0.22 | 1.81E-10 |
|  | LOC101261704 | fumarylacetoacetase | -0.29 | 1.20E-37 |
|  | LOC101246092 | p-coumaroyl quinate/shikimate 3-hydroxylase | -0.36 | 8.47E-04 |
|  | LOC101243656 | phenylalanine ammonia-lyase 6 | -0.42 | 1.47E-18 |
|  | LOC101256510 | beta-glucosidase 40 | -0.65 | 5.62E-06 |
|  | LOC101255909 | 4-coumarate--CoA ligase-like 6 | -0.91 | 6.72E-05 |
|  | PAL5 | phenylalanine ammonia-lyase 5 | -0.92 | 5.97E-09 |
|  | CHS1 | chalcone synthase | -0.96 | 2.58E-45 |
|  | LOC101261193 | anthocyanidin 3-O-glucosyltransferase 5 | -1.23 | 5.75E-04 |
|  | LOC101265461 | histidine decarboxylase-like | -1.29 | 2.78E-04 |
|  | LOC101266674 | probable aminotransferase TAT2 | -1.30 | 2.83E-17 |
|  | CHI1 | chalcone--flavonone isomerase 1 | -1.61 | 5.14E-23 |
|  | LOC101257526 | beta-glucosidase 18 | -1.63 | 7.79E-43 |
|  | LOC101263431 | tryptophan decarboxylase TDC1-like | -1.66 | 7.79E-07 |
|  | LOC101265606 | berberine bridge enzyme-like 15 | -1.72 | 2.10E-07 |
|  | LOC101247834 | caffeic acid 3-O-methyltransferase-like | -1.76 | 1.02E-10 |
|  | F3H | flavanone 3-dioxygenase | -1.88 | 3.60E-16 |
|  | CHS2 | chalcone synthase | -2.25 | 1.61E-42 |
|  | TAP2 | Suberization-associated anionic peroxidase 2 | -2.56 | 2.19E-06 |
|  | F3'5'H | flavonoid 35 hydroxylase | -2.77 | 8.36E-43 |
|  | LOC101253862 | primary amine oxidase 1 | -3.29 | 2.08E-04 |
|  | TPX2 | peroxidase | -3.32 | 1.80E-05 |
|  | LOC101264847 | histidine decarboxylase-like | -3.50 | 9.27E-12 |
|  | LOC100191129 | anthocyanin acyltransferase | -4.25 | 5.85E-19 |
|  | LOC101248236 | peroxidase 44-like | -5.34 | 3.32E-11 |
| COR | LOC101256510 | beta-glucosidase 40 | -0.14 | 0 |
|  | LOC101262081 | linoleate 13S-lipoxygenase 2-1, chloroplastic | -0.15 | 2.99E-04 |
|  | LOC101265606 | berberine bridge enzyme-like 15 | -0.18 | 1.73E-05 |
|  | TAP2 | Suberization-associated anionic peroxidase 2 | -0.27 | 3.33E-07 |
|  | F3H | flavanone 3-dioxygenase | -0.33 | 1.20E-37 |
|  | LOC101257526 | beta-glucosidase 18 | -0.61 | 1.17E-09 |
|  | LOC101253032 | caffeoyl-CoA O-methyltransferase 5 | -0.62 | 2.53E-04 |
|  | LoxC | lipoxygenase | -0.65 | 9.43E-04 |
|  | LOC101266674 | probable aminotransferase TAT2 | -0.86 | 3.01E-09 |
|  | CHI1 | chalcone--flavonone isomerase 1 | -0.91 | 3.54E-10 |
|  | LOC101263431 | tryptophan decarboxylase TDC1-like | -0.93 | 0.001899804 |
|  | F3'5'H | flavonoid 35 hydroxylase | -0.97 | 6.37E-11 |
|  | LOC101247834 | caffeic acid 3-O-methyltransferase-like | -1.25 | 3.33E-07 |
|  | LOC101265461 | histidine decarboxylase-like | -1.26 | 2.99E-04 |
|  | CHS2 | chalcone synthase | -1.36 | 2.53E-04 |
|  | LOC101261704 | fumarylacetoacetase | -1.44 | 1.73E-05 |
|  | CHS1 | chalcone synthase | -1.51 | 3.67E-13 |
|  | LOC101253556 | agmatine hydroxycinnamoyltransferase 1-like | -1.54 | 0.00215708 |
|  | LOC101253862 | primary amine oxidase 1 | -2.12 | 0.00254348 |
|  | LOC112940337 | lignin-forming anionic peroxidase-like | -2.12 | 0.0074072 |
|  | TPX2 | peroxidase | -3.17 | 1.89E-05 |
|  | LOC101248236 | peroxidase 44-like | -5.34 | 3.62E-11 |
